# Engineering inducible biomolecular assemblies for genome imaging and manipulation in living cells

**DOI:** 10.1038/s41467-022-35504-x

**Published:** 2022-12-24

**Authors:** Qin Peng, Ziliang Huang, Kun Sun, Yahan Liu, Chi Woo Yoon, Reed E. S. Harrison, Danielle L. Schmitt, Linshan Zhu, Yiqian Wu, Ipek Tasan, Huimin Zhao, Jin Zhang, Sheng Zhong, Shu Chien, Yingxiao Wang

**Affiliations:** 1 Department of Bioengineering, Institute of Engineering in Medicine, University of California, La Jolla, CA 92093-0435 USA; 2 Institute of Systems and Physical Biology, Shenzhen Bay Laboratory, Shenzhen, 518132 P. R. China; 3 Institute of Cancer Research, Shenzhen Bay Laboratory, Shenzhen, 518132 P. R. China; 4 Department of Pharmacology, University of California, La Jolla, CA 92093-0435 USA; 5 Department of Biochemistry, University of Illinois at Urbana-Champaign, Urbana, IL 61801 USA; 6 Department of Chemical and Biomolecular Engineering, University of Illinois, Urbana-Champaign, Urbana, IL 61801 USA; 7 Department of Medicine, University of California, La Jolla, CA 92093-0435 USA

**Keywords:** Molecular engineering, Fluorescence imaging, Epigenetics, Chromatin, Gene regulation

## Abstract

Genome architecture and organization play critical roles in cell life. However, it remains largely unknown how genomic loci are dynamically coordinated to regulate gene expression and determine cell fate at the single cell level. We have developed an inducible system which allows Simultaneous Imaging and Manipulation of genomic loci by Biomolecular Assemblies (SIMBA) in living cells. In SIMBA, the human heterochromatin protein 1α (HP1α) is fused to mCherry and FRB, which can be induced to form biomolecular assemblies (BAs) with FKBP-scFv, guided to specific genomic loci by a nuclease-defective Cas9 (dCas9) or a transcriptional factor (TF) carrying tandem repeats of SunTag. The induced BAs can not only enhance the imaging signals at target genomic loci using a single sgRNA, either at repetitive or non-repetitive sequences, but also recruit epigenetic modulators such as histone methyltransferase SUV39H1 to locally repress transcription. As such, SIMBA can be applied to simultaneously visualize and manipulate, in principle, any genomic locus with controllable timing in living cells.

## Abstract (web summary)

Imaging non-repetitive loci in living cells remains challenging. Here, the authors engineered an inducible system whereby biomolecular assemblies can be guided to specific genomic loci by a nuclease-defective Cas9, allowing the simultaneous imaging and manipulation of the loci.

## Introduction

It has become clear that oligomers can assemble and condensate into nano-scale and functional membraneless organelles^1–3^. In the nucleus, these condensed biomolecular assemblies (BAs) may interact with chromatin and regulate genome architecture and gene expression^4^. For example, heterochromatin protein 1α (HP1α) is a major component of constitutive heterochromatin, with important roles in shaping chromatin structure and controlling genome stability and gene expression^5,6^. HP1α has the capability to condense in vitro and in vivo, through weak hydrophobic interactions driven by the intrinsically disordered region (IDR) in the N-terminal extension and hinge domain^7–9^. The high local concentration of HP1α can promote BA formation at the genomic loci, facilitated by homo- and hetero-interactions involving proteins, RNAs, and DNAs^10^. Specific buffer conditions and environmental co-factors at the genomic regions may also promote chromatin condensates^9^. In fact, methyl-CpG binding protein 2 (MeCP2) can form condensates in vitro only in the presence of DNA or nucleosomes^10,11^.

Structurally, HP1α consists of an N-terminal chromodomain (CD) for its binding to K9-trimethylated histone H3 (H3K9me3), a middle positively-charged hinge domain (HD) for DNA and RNA binding, and a C-terminal chromo shadow domain (CSD) for self-association^7^. HP1α can directly interact with and recruit SUV39H1/2 and SETDB1^12,13^, histone methyltransferases that deposit the transcriptionally suppressive mark H3K9me3, to facilitate self-propagation and sequential methylation of adjacent nucleosomes, for the recruitment of more HP1α molecules at the local region^14,15^. These unique characteristics of HP1α have inspired us to examine whether HP1α can be utilized to trigger BA formation for genomic locus labeling, especially for non-repetitive loci where only one copy of dCas9-sgRNA complex can bind with a single sgRNA. In addition, the transcription suppression capability of HP1α may also allow local gene silencing at the labeled loci in living cells.

To visualize genomic loci in living cells, various catalytically inactive CRISPR/Cas9 (dCas9)-based labeling systems have been developed^16–21^. Among those designs, the relatively low signal-to-noise-ratio (SNR) at specific genomic loci, particularly those with non-repetitive sequences, is a major challenge to overcome. Currently, there are several strategies to amplify the signals and enhance SNR for locus labeling, including bimolecular fluorescence complementation (BiFC)^16,22^, tandem repeats of MS2 stem loop^20,23^ and multiple repeats of SunTag^16,21,22^, typically integrated with the dCas9-sgRNA system. For the labeling of non-repetitive loci, as there is only one copy of anchoring site for a dCas9-sgRNA complex at a genomic locus, the locus-specific labeling using a single sgRNA is generally inefficient. Currently, multiple sgRNAs targeting neighboring regions of the target locus are still required for non-repetitive locus labeling^17,20,24–26^, which typically result in increased complexity, reduced efficiency, and higher risk of off-target labeling.

Tools have also been developed for locus-specific perturbation of gene regulations. For example, CRISPRa can activate gene expression by fusing transcription factors such as VP64 to dCas9 to target specific loci^27–30^. CRISPRi can suppress gene expression by fusing repressors such as KRAB or HDAC to dCas9 to change the epigenetic landscape surrounding the genomic loci^27,31^. Researchers further incorporated tandem repeats of SunTag to enhance the power of the dCas9 for gene regulation^32^, by recruiting multiple copies of the genetic and epigenetic effectors to target loci through the high-affinity association between the 19-amino-acid SunTag and its corresponding single chain variable fragments (scFv) antibody. Indeed, different regulators, such as VP64^32^, DNMT3A^33^, P65-HSF1^34^, and P300^35^, can be fused with scFv, which can bind to the tandem repeats of SunTag fused to dCas9 and induce efficient genetic or epigenetic manipulation at the desired genomic loci.

Here we report the development of a SIMBA (simultaneous imaging and manipulation with biomolecular assemblies) system, which is capable of labeling genomic loci containing non-repetitive sequences with a single sgRNA and suppressing the local transcription. SIMBA can induce BAs at target genomic loci to amplify the signals for locus labeling and manipulation via the multilayer and multivalent interactions between HP1α and tandem repeats of SunTag. SIMBA can also be guided by endogenous transcription factors such as NFAT1 to reprogram the innate gene expression profile of cells. We further demonstrate that SIMBA-mediated gene suppression is mainly due to the local recruitment of the histone methyltransferase SUV39H1/2, suggesting an epigenetic remodeling role of SIMBA in regulating genomic loci.

## Results

### SIMBA enables labeling of repetitive genomic loci

**Fig. 1:**
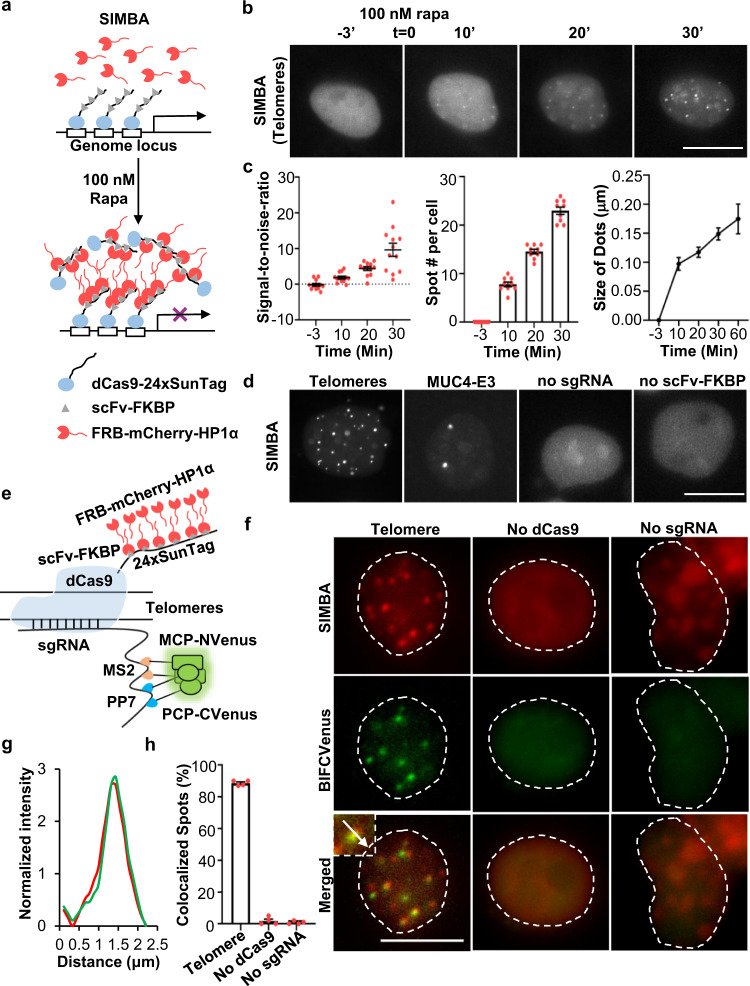
The design of SIMBA and its application to label genomic loci with repetitive sequences. **a** Schematic diagram of SIMBA, with its components including dCas9-24xSunTag, scFv-FKBP, and FRB-mCherry-HP1α. Upon rapamycin addition, HP1α can be recruited to target genomic loci and forms puncta. **b** Representative time-lapse images showing puncta targeted at telomeres formed upon 100 nM rapamycin addition. Scale bar, 10 μm. **c** Quantified signal-to-noise ratio (SNR) (Left), the numbers of labeled puncta per cell detected at different time points (Middle), and the size change in time upon the rapamycin treatment (Right) (*n* = 5, 7, 8, 9, 12 nuclei). SNR number was listed in Supplementary Data 1. Error bars, mean ± SEM. **d** Representative images showing puncta formed at the *Telomeres* or *MUC4* locus after 100 nM rapamycin treatment for 2 hrs in HEK293T cells. No sgRNA or scFv-FKBP groups serve as the negative control. Scale bar, 10 μm. **e** Schematic diagram showing double labeling of telomeres by using BiFC and SIMBA. **f** Images of telomere loci labeling with both BiFC and SIMBA in HEK293T cells upon rapamycin treatment. No dCas9 and sgRNA groups serve as the negative controls. The images were from a single slice, with possibly a slight shift in the z-plane for the images. Scale bar, 10 μm. **g** Normalized intensity curves from SIMBA (red) and BiFC (green) at a highlighted locus in (**f**). **h** The percentage of labeled telomere loci with co-localized SIMBA (red) and BiFC (green). Error bars, mean ± SEM. *n* = 4 biologically independent experiments for all three groups. Source data are provided as a Source Data file.

To amplify the signals for genomic locus labeling, we have developed SIMBA, which is a locus-specific inducible BA formation system based on the multilayer and multivalent interactions between HP1α and SunTag tandem repeats (Fig. 1a). In SIMBA, FRB-mCherry-HP1α can be recruited to dCas9 fused with 24 repeats of SunTag (dCas9-24xSunTag), mediated by the fusion protein scFv-FKBP, which can, on one hand, constitutively bind SunTag with high affinity (the scFv arm), and, on the other hand, bind FRB upon rapamycin induction (the FKBP arm). All the components are contained in the nucleus by the fused nuclear localization signal (NLS) to enhance the targeting efficiency. We first tested SIMBA for the labeling of the repetitive *telomere* satellite sites with one telomere-targeting sgRNA (*Telo*sgRNA) in HEK293T cells (Fig. 1a). In the absence of the FKBP-FRB heterodimerization inducer rapamycin, FRB-mCherry-HP1α showed generally uniform distribution in the nucleus^36–38^. Upon induction with 100 nM rapamycin, the puncta highlighted by FRB-mCherry-HP1α gradually became visible (Fig. 1b). These puncta were initiated at the *telomere* sites within 10 min of rapamycin addition and stabilized after 30 min, showing an increase in number and size of the labeled puncta, as well as their signal-to-noise ratio (SNR) (Fig. 1c)^22^. In contrast, in the negative control cells lacking *Telo*sgRNA, only mCherry aggregations occurred in nucleolus-like structures, typical for non-specific clustering of molecular aggregates in the nucleolus (Supplementary Fig. 1a)^17,20^. These results suggest that SIMBA can specifically label genomic loci as designed. We also examined SunTag-HP1α system where scFv is directly fused to HP1α without the inducible FKBP-FRB dimerization, and constitutive BAs were observed at the targeted loci (Supplementary Fig. 1b), although the labeling timing cannot be controlled in this case. Furthermore, the total fluorescent intensity on the puncta upon rapamycin treatment and the basal mean mCherry intensity were measured. The total puncta signal serves as a function of overall basal scFv-mCherry-HP1α concentration. The results showed that there was a positive correlation between total puncta signal and mean mCherry signal (Supplementary Fig. 1c), and the absolute fluorescence intensity increased significantly on the puncta, but not for nuclear background or nucleolus (Supplementary Fig. 1d). These factors led to most of labeled puncta achieving SNR above 10, indicating high labeling efficiency and specificity (Supplementary Fig. 1e). To demonstrate the versatility of our method, we further applied SIMBA to label the repetitive exon 3 region of human *MUC4* gene (*MUC4-E3*)^39^; SIMBA clearly led to two labeled puncta in a single nucleus of a living cell, but not in negative controls missing either sgRNA or scFv-FKBP (Fig. 1d), which is consistent with previous observations^20^.

To further verify the specificity of SIMBA in labeling genomic loci, we developed an orthogonal labeling method complementary to SIMBA, by engineering the sgRNA to carry both MS2 and PP7 stem loops^19^, which can recruit the split pair of yellow fluorescent protein Venus (N-Venus and C-Venus) via the MS2 and PP7 coat proteins MCP and PCP, respectively (Fig. 1e). As such, the dCas9/sgRNA complex can bring together the non-fluorescent MCP-fused N-Venus and PCP-fused C-Venus to form complemented fluorescent Venus only at the target loci via biomolecular fluorescence complementation (BiFC). Indeed, this BiFC system, despite relatively higher background noise, allowed the labeling of repetitive *MUC4-E3*^39^, similar to the labeling of SIMBA (Supplementary Fig. 2a–c).

To evaluate the accuracy of these complementary labeling approaches in the same cells, we integrated the BiFC system and SIMBA by adopting the sgRNA with MS2-PP7 loops, along with N-Venus-MCP and C-Venus-PCP co-transfected in the same cells (Fig. 1e). This enabled us to track the labeling from both SIMBA (represented by mCherry) and sgRNA-BiFC (represented by Venus). The identified puncta by mCherry and Venus clearly co-localized in the same cells when targeting either the *telomere* or *MUC4-E3* loci, in contrast to the diffusive signals in the control groups without sgRNA or dCas9 (Fig. 1f–h and Supplementary Fig. 2d, e). These results suggest that SIMBA can label the target genomic loci with high precision. We have also generated the corresponding lentiviral constructs including all the components of SIMBA, and further generated the SIMBA stable cell line using human U2OS cells. Upon rapamycin addition, SIMBA puncta targeting *MUC4-E3* loci can be clearly induced in these cells (Supplementary Fig. 2f). These cells were cultured after more than five passages since cell sorting, suggesting the stable cell line is compatible for long-term imaging. Non-specific and faint puncta have also been observed during our tests with SIMBA, even before rapamycin treatment (Supplementary Fig. 2f), which could be due to the chromatin binding property of HP1α. In some cells, the puncta observed after rapamycin were more than the theoretical copy number, which could be due to genome replication although these loci would appear as doublets in G2 phase^40,41^. To distinguish specific SIMBA-labeled puncta from the non-specific ones, the cut-off threshold of SNR = 10 was applied to determine the specific puncta based on the strong signal amplification nature of SIMBA (Supplementary Fig. 1e), which can effectively distinguish non-specific labeling as observed in Supplementary Fig. 2f.

To titrate the HP1α molecule amount recruited at the dCas9-targeted locus for BA formation, we varied the SunTag repeats fused to dCas9 from 24x to 4x, 2x, or 1x (Supplementary Fig. 3). The results showed that there is a proportional reduction in BA formation (targeting *MUC4-E3*) as the number of SunTag repeats decreases (Supplementary Fig. 3). Particularly, clear BAs can no longer be observed in the 1x or 2x SunTag-fused dCas9 groups upon rapamycin induction, although BAs can form efficiently in the 24x SunTag group and with reduced efficiency in the 4x SunTag group (Supplementary Fig. 3a, b, Supplementary Data 1).

We have further identified the HP1α domains that are important for BA formation, by engineering HP1α mutants with the truncation of N-terminus and chromo domain (delta1: 1–72)^42^, hinge domain (delta2: 81–105)^43^, or chromo shadow domain (delta3: 121–179)^44^. Both the HP1α delta1 and delta2, but not the delta3, allow BA formation (Supplementary Fig. 3c). These results suggest that the chromo shadow domain, but not chromo domain or hinge domain, is crucial for SIMBA-mediated HP1α BA formation, highlighting the critical role of interactions between HP1α molecules mediated by the chromo shadow domain.

Interestingly, when we replaced HP1α in the SIMBA system with the N-terminus of the Fused in Sarcoma protein (FUS_N_), a widely-used IDR, robust BA formation was also observed upon rapamycin induction (Supplementary Fig. 3d), suggesting that mechanistically, multivalent interactions from FUS IDR and HP1α chromo shadow domain are the key for BA formation in SIMBA. We further engineered a dCas9-24xSunTag-GFP construct and co-transfected with SIMBA system for *MUC4* locus labeling. We observed the recruitment of dCas9-24xSunTag-GFP molecules at the target regions, which was likely recruited by the self-aggregation of HP1α molecules, as dCas9-24xSunTag-GFP itself did not cause puncta formation without the rapamycin induction (Supplementary Fig. 3e). This result suggests that the self-aggregation of HP1α molecules in SIMBA can trigger the recruitment of more dCas9 copies at the target locus sites.

We also examined the biophysical property of BAs by applying 1,6-hexanediol (1,6-HD), which disrupts the weak multivalent interactions of proteins containing IDRs^8^. Upon the addition of 2.5% 1,6-HD after the removal of rapamycin, the puncta labeled by *MUC4*-targeting SIMBA in HEK293T cells gradually faded away within 15 min (Supplementary Fig. 4a, b). In contrast, without 1,6-HD treatment, the SIMBA signals remained strong, even after rapamycin washout, for > 12 hrs in most cells (Supplementary Fig. 4c, d).

We further conducted fluorescence recovery after photobleaching (FRAP) experiments to examine whether there is material exchange of SIMBA components between the puncta and the surrounding environment^7,8^. Indeed, the mCherry fluorescence of SIMBA puncta showed significant recovery after photobleaching (Supplementary Fig. 4e, f), revealing the dynamic characteristics of these BAs.

### SIMBA enables labeling of non-repetitive genomic loci

**Fig. 2:**
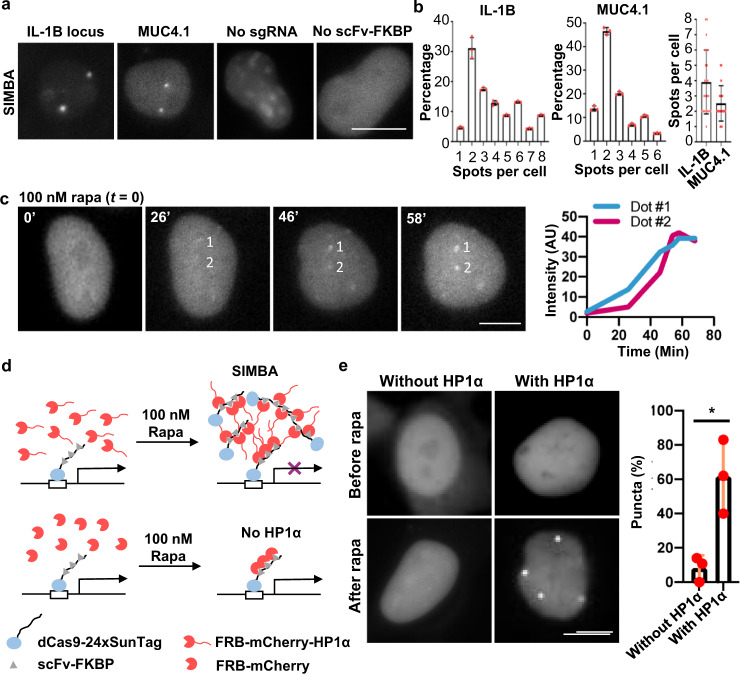
SIMBA can label non-repetitive loci. **a** Images of HEK293T cells expressing dCas9-24xSunTag, scFv-FKBP, FRB-mCherry-HP1α, and a single sgRNA for the labeling of non-repetitive *IL-1B* or *MUC4.1* locus after rapamycin treatment. Scale bar, 5 μm. **b** Bar graphs show the distributions of three independent experiments (Left and Middle, *n* = 3 biologically independent experiments) and the averages (Right) of puncta per cell detected by SIMBA labeling of *IL-1B* and *MUC4.1* loci. *n* = 23 cells for *IL-1B* group, *n* = 29 cells for *MUC4.1* group. Error bars, mean ± SD. **c** Time-lapse images showing droplet formation at *IL-1B* locus following 100 nM rapamycin treatment. The corresponding SNR numbers for each time point were calculated and listed in Supplementary Data 1. Scale bar, 5 μm. **d** Schematic drawing depicting the mechanism of non-repetitive locus labeling by SIMBA with or without HP1α. **e** There was no puncta at loci after rapamycin treatment in the negative group without HP1α. *n* = 3 biologically independent experiments, unpaired two-tailed Student’s *t*-test, *p* = 0.0152. *, *p* < 0.05. Error bars, mean ± SD. Scale bar, 5 μm. Source data are provided as a Source Data file.

We continued to examine whether SIMBA can be applied to label non-repetitive genomic loci in living cells with a single sgRNA. HEK293T cells were transfected with SIMBA along with a sgRNA targeting either non-repetitive *IL-1B* or *MUC4.1*^20^. The genome of HEK293T cells has been reported to contain two to three copies of the sgRNA-targeted regions for *IL-1B* and *MUC4*^45^. Consistently, we observed 2–4 labeled puncta per cell for both *IL-1B* (3.9 ± 0.44 puncta per cell, mean ± SEM) and *MUC4.1* (2.5 ± 0.21 puncta per cell, mean ± SEM), while no clear puncta were detected in the control groups where either sgRNA or scFv-FKBP was absent (Fig. 2a, b). Time-course images further revealed that mCherry intensity of the puncta increased gradually and stabilized within 1 hr of rapamycin treatment (Fig. 2c). In a control system where HP1α was absent from the SIMBA system, no labeled puncta were observed before or after rapamycin treatment (Fig. 2d, e). These results revealed the crucial role of HP1α in SIMBA puncta formation, possibly via the multivalent property of HP1α^7^. Interestingly, we noticed a slightly weakened nuclear localization of mCherry in the absence of HP1α (Fig. 2e), reflecting the role of the nuclear localization signal within HP1α as previously reported^46^. In contrast, attaching multiple repeats of MS2-PP7 loops in the sgRNA, although previously demonstrated capable of labeling repetitive loci, was not able to label non-repetitive loci (Supplementary Fig. 5). As such, our results demonstrated that SIMBA is able to label non-repetitive genomic loci in living cells, where HP1α plays a critical role.

### SIMBA can suppress gene expression at both synthetic and endogenous genomic loci

**Fig. 3:**
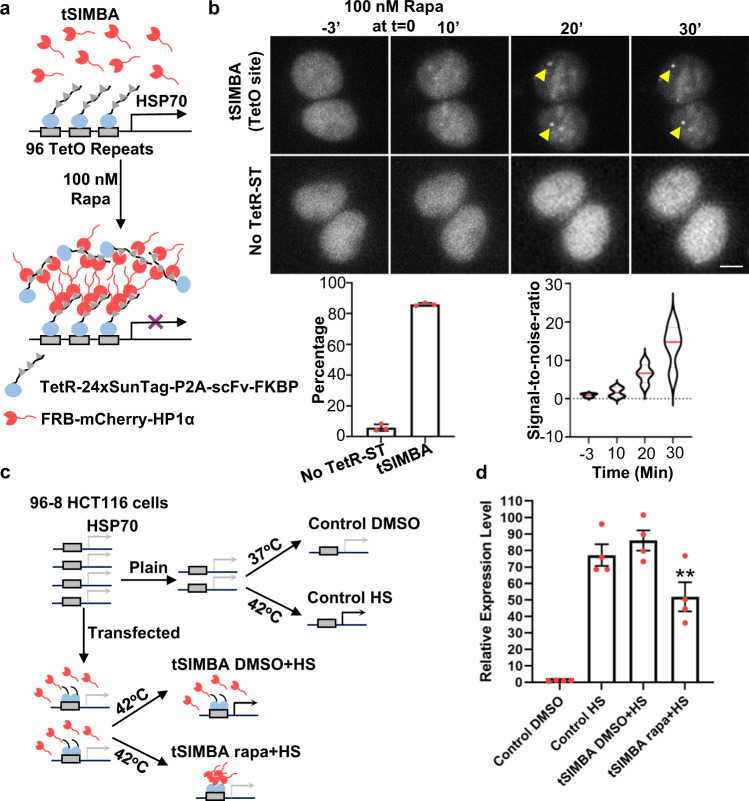
SIMBA enables puncta formation at a synthetic site inserted in the genome to suppress local gene expression. **a** Schematic diagram of tSIMBA, with its components including TetR-24xSunTag-P2A-scFv-FKBP and FRB-mCherry-HP1α. Upon rapamycin addition, HP1α is recruited to the 96 x TetO site in engineered HCT116 cells to regulate heat shock (HS)-induced gene expression. **b** Top panel: Time-lapse images showing puncta formation at the single engineered TetO site (Yellow arrow) in the genome of a HCT116 cell following 100 nM rapamycin treatment. In contrast, no TetO labeling can be observed in the negative control group without TetR-24xSunTag-P2A-scFv-FKBP. Bottom panel: the percentage of the cells with puncta and SNR from top panel were quantified. SNR number was listed in Supplementary Data 1. *n* = 3 biologically independent experiments for both groups. Scale bar, 5 μm. **c** Schematic diagram of the experimental design in evaluating the tSIMBA effect on the HS-induced gene expression of *HSPA1A* next to the engineered TetO site. **d** The relative expression level of *HSPA1A* gene in response to HS in 96-8 HCT116 cells with or without tSIMBA (examined by qPCR). Control HS and DMSO groups represent the naïve 96-8 HCT116 cells with or without HS stimulation, respectively. tSIMBA DMSO + HS and tSIMBA rapa+HS represent the HS-induced 96-8 HCT116 cells expressing tSIMBA, pre-treated by DMSO or rapamycin, respectively (unpaired two-tailed Student’s *t*-test, *p* = 0.019005 (tSIMBA DMSO + HS Vs. tSIMBA rapa+HS), *n* = 4 biologically independent experiments). *p < 0.05. Error bars, mean ± SEM. Source data are provided as a Source Data file.

As HP1α-recruitment at promoter region can induce heterochromatin formation and suppress gene expression^12^, we examined whether SIMBA can also suppress gene expression when targeted at a specific locus. We first targeted SIMBA to a 96-repeat tetracycline operator (TetO) inserted at one allele of the heat shock protein 70 (*HSP70*) locus (upstream of *HSPA1A* on Chr6) in an engineered HCT116 cell line (96-8 HCT116)^47^ using TetR-24xSunTag (tSIMBA, Fig. 3a). Upon rapamycin stimulation, one punctum can be clearly observed and gradually appeared in each transfected cell, which was not observed in cells without TetR-24xSunTag (Fig. 3a, b). The labeling efficiency of tSIMBA is relatively high and stable for long periods (Supplementary Fig. 5a, b, d). Thus, SIMBA can target engineered genomic regions, e.g., the TetO array inserted at *HSPA1A* in HCT116 cells.

We further studied the gene suppression effect of SIMBA in the engineered HCT116 cells, by pretreating the cells with either rapamycin or DMSO (as control) for 1 hr, and then applying heat shock (HS) (42 °C for 30 min) to activate the *HSPA1A* locus. RNAs were then extracted for qPCR analysis to examine the effect of SIMBA on *HSP70* gene expression (Fig. 3c and Supplementary Fig. 6c). Our results showed that the HS-induced expression of *HSP70* gene was significantly suppressed by SIMBA, but not in the control groups (without transfection or rapamycin) (Fig. 3d). These results suggest that SIMBA can be targeted at engineered sites in the genome to suppress the local transcription.

**Fig. 4:**
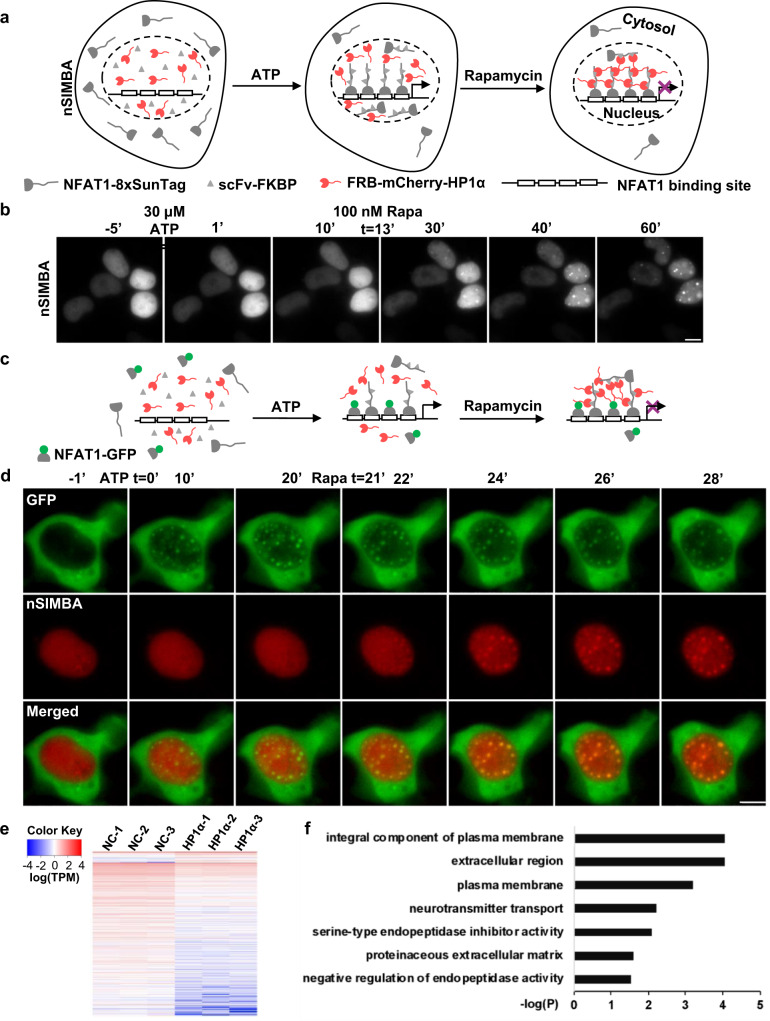
SIMBA enables puncta formation and gene manipulation at the endogenous NFAT1 binding sites in the genome. **a** nSIMBA consists of three components, NFAT1-8xSunTag, scFv-FKBP, and FRB-mCherry-HP1α. Upon ATP treatment, NFAT1-8xSunTag can translocate into nucleus and target NFAT1 binding motifs in the genome together with scFv-FKBP. Upon rapamycin stimulation, FRB-mCherry-HP1α can be further recruited to the genome sites targeted by NFAT1 and promote the puncta formation. **b** Time-lapse images show the labeling of NFAT1 binding sites through the nSIMBA system in HEK293T cells, upon the sequential treatments of 30 μM ATP and 100 nM rapamycin. Scale bar, 5 μm. **c**, **d** Diagram (**c**) and live cell images (**d**) showing the sequential recruitment of NFAT1-GFP and nSIMBA at the target genomic loci, upon ATP and rapamycin stimulation. Scale bar, 5 μm. **e** Heat-map shows differential gene expression between groups with (HP1α) or without HP1α (NC). **f** GO-term enrichment analysis of significantly altered genes from RNA-seq results by using DAVID. The bars show the functional gene groups that are downregulated by HP1α. The y axis shows the top seven enriched GO terms and the *x* axis shows the enrichment significance of Benjamini corrected *P*-values.

Nuclear factor of activated T-cells (NFAT) is a family of transcription factors critical in immune responses^48^. NFAT1, one of the most studied NFAT family members, can bind to a variety of endogenous loci in the genome (Supplementary Fig. 7). NFAT1 can hence provide a targeting mechanism for us to study the labeling and suppressive effects of SIMBA on multiple genomic loci. We fused NFAT1 to tandem repeats of SunTag, which is expected to target SIMBA to the NFAT1-binding loci throughout the genome upon rapamycin treatment (nSIMBA, Fig. 4a). As nuclear shuttling and DNA binding of NFAT1 are tightly regulated by intracellular Ca^2+^ signaling^49^, 30 μM ATP was applied to trigger the Ca^2+^ signaling and stimulate nuclear translocation of NFAT1 (Supplementary Fig. 8). Rapamycin was then added to the cells to induce the punctum formation at NFAT1 binding sites, leading to multiple labeled puncta (Fig. 4b). To further demonstrate the specificity of SIMBA in labeling the NFAT1 binding sites in the genome, we co-transfected HEK293T with both nSIMBA and NFAT1-GFP, which can highlight the endogenous NFAT1 binding sites in parallel with distinct colors (Fig. 4c). NFAT1-GFP accumulated first at the loci in the nucleus within 10 min upon ATP treatment, followed by the nSIMBA-induced puncta (mCherry) formed within 5 min upon rapamycin treatment, which co-localized well with the NFAT1-GFP puncta (Fig. 4d). These results demonstrate that SIMBA can label the endogenous NFAT1 binding sites with high accuracy. Additionally, we observed dynamic fusion among neighboring puncta (Supplementary Video 1), possibly reflecting the interactions of SIMBA puncta^50^.

We then examined the effect of nSIMBA on target gene expression (Supplementary Fig. 9). Cells expressing nSIMBA were stimulated by ATP and rapamycin as described above and harvested for transcriptomic analysis by RNA-seq at 6 hrs after rapamycin stimulation (Supplementary Fig. 9a). We observed suppression in a large proportion of NFAT1-regulated genes (395 genes downregulated, 28 genes upregulated) as compared with the negative control where HP1α was absent from nSIMBA (Fig. 4e, Supplementary Data 2). The most enriched terms in Gene Ontology (GO) of nSIMBA suppression were related to plasma membrane and extracellular matrix (Fig. 4f), consistent with the reported functions of NFAT1^51^.

### SIMBA-induced gene suppression is mainly due to recruitment of SUV39H1 to the target loci

**Fig. 5:**
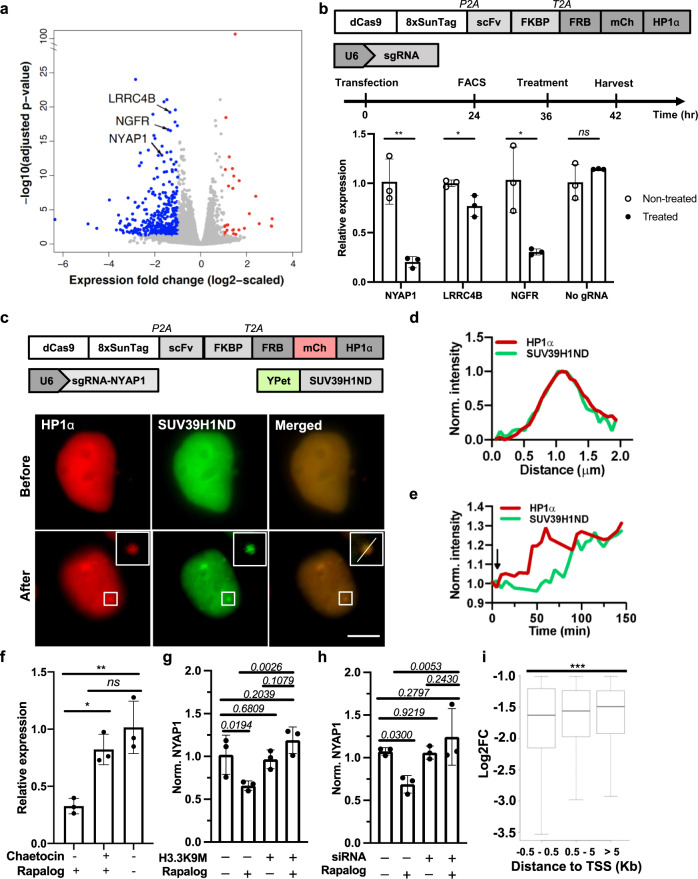
SIMBA recruits histone methyltransferase to regulate local transcription at target genomic sites. **a** Volcano plot of HP1α-mediated transcriptomic changes at NFAT1 binding sites (Benjamini corrected *P*-value, *P*_adj_ < 0.05). *NYAP1*, *NGFR*, and *LRRC4B* selected for following validation assays. Differentially regulated genes shown in blue (downregulated) and red (upregulated). **b** The CRISPR/dCas9 system targets the three endogenous genes in HEK293T cells. qPCR results show the relative expression (n = 3 biologically independent experiments, unpaired two-sided Student’s *t* test. *NYAP1*: *p* = 0.0039; *LRRC4B*: *p* = 0.0244; *NGFR*: *p* = 0.0191; No gRNA: *p* = 0.2662.) **c** Live cell imaging shows co-localization of HP1α and SUV39H1 N-terminal domain (SUV39H1ND) at *NYAP1* locus before and after rapalog treatment (insets: enlarged images of the indicated BAs with enhanced clarity by applying more stringent brightness/contrast settings). Scale bar, 5 μm. **d** The puncta highlighted by the white box in (**c**) was quantified based on normalized intensities. **e** Dynamic fluorescence intensity change of HP1α (red) and SUV39H1ND (green) upon rapamycin (black arrow). Average intensity values of HP1α or SUV39H1ND at different time points were normalized to that of the time points before rapamycin. **f** *NYAP1* gene expression determined by qPCR in response to chaetocin treatment (500 nM, 6 hrs), with or without HP1α BA formation. (*n* = 3 biologically independent experiments. One-way ANOVA with Tukey’s multiple comparison test; *p = 0.0202; **p = 0.0043; *ns*, *p* = 0.3535.). **g**, **h** Effects of H3.3K9M (**g**) and SUV39H1/2 knockdown (**h**) on SIMBA-induced gene suppression. Target gene: *NYAP1*. *n* = 3 biologically independent experiments, two-sided Fisher’s LSD test. *p* values are indicated in the figure. **i** The correlation between the distance of NFAT1 binding sites to TSS and fold change of the downregulated genes from nSIMBA RNA-seq data. Downregulated genes were divided into 3 categories based on the distance between nSIMBA binding sites to TSS. Standard boxplots, *n* = 145, 107, 97 genes, respectively. *** Two-sided Kruska–Wallis test, *p* = 4.9 × 10^−7^. Error bars, mean ± SD. *ns*, not significant. Source data are provided as a Source Data file.

We further selected three top-ranked genes downregulated by nSIMBA with their promoters located close to the predicted NFAT1 binding sites (see Methods section): *NYAP1*, *LRRC4B*, and *NGFR* (Fig. 5a, Supplementary Fig. 9b). We applied the dCas9-guided SIMBA system to target these loci and verified the locus-specific gene suppression. The qPCR results showed that all three genes can be significantly suppressed by SIMBA upon rapamycin treatment, whereas no significant suppression (based on *NYAP1* levels) was detected with rapamycin treatment when sgRNA was absent (Fig. 5b). These results suggest that SIMBA can be applied to not only label genomic loci but also manipulate local gene expression. Interestingly, with 1x SunTag in SIMBA, although there was no punctum formation in this case, gene suppression can still be observed, suggesting that puncta formation is not required for SIMBA-mediated gene repression (Supplementary Fig. 10).

We further investigated the underlying mechanism by which SIMBA causes endogenous gene suppression. HP1α has been shown to directly interact with and recruit the repressive H3K9-specific methyltransferases (HMTs) SUV39H1/2 and SETDB1 to facilitate self-propagation and sequential methylation of adjacent nucleosomes^12,38^. We hence hypothesized that the SIMBA-mediated HP1α assemblies would also recruit SUV39H1/2 to the target loci for gene suppression. To visualize whether there is co-localization of HP1α and SUV39H1 in single cells, we deployed a system consisting of (i) the engineered human U2OS 2-6-3 cell line, which carries repetitive arrays of TetO sites on chromosome 1 (1p36)^52^, (ii) the fusion protein TetR-EGFP-HP1α, which can be recruited to the TetO sites in U2OS 2-6-3 cells, and (iii) the fusion protein of mCherry and the SUV39H1 N-terminal domain (mCherry-SUV39H1ND)^53^, which is the domain responsible for HP1α binding^54^ (Supplementary Fig. 11a). We observed that TetR-EGFP-HP1α formed a bright punctum at the repetitive TetO sites, which co-localized well with mCherry-SUV39H1ND (Supplementary Fig. 11b), suggesting that SUV39H1 can be recruited to the SIMBA-mediated biomolecular assemblies of HP1α in living cells. We then tracked the recruitment of SUV39H1 to the SIMBA-mediated HP1α when targeting the endogenous genomic loci, using *NYAP1* locus as an example. The SIMBA-labeled *NYAP1* loci (mCherry in Fig. 5c) clearly appeared in cells upon rapamycin stimulation, accompanied by a delayed accumulation of YPet-SUV39H1ND at the same location (YPet in Fig. 5c–e). On the other hand, when cells were pre-treated with chaetocin, a histone methyltransferase inhibitor, the gene suppression effect of SIMBA upon rapamycin was almost abolished (Fig. 5f), although punctum formation can still be observed (Supplementary Fig. 11c). Further tests with co-transfection of histone H3.3 mutant H3.3 K9M, which can trap and neutralize methyltransferases to inhibit H3K9 methylation^55^, as well as the knockdown of SUV39H1/2 via siRNA, also showed significant reduction of the gene suppression caused by SIMBA (Fig. 5g–h). These results suggest that the SIMBA-mediated gene repression is mainly through SUV39H1/2 methyltransferases. Overall, our results suggest that SIMBA can trigger the recruitment of SUV39H1/2 at the targeted loci, leading to the local histone methylation and gene suppression^12^. This is consistent with earlier reports that HP1α chromo shadow domain can recruit SUV39H1 via its N-terminal domain to suppress local transcriptions^54^. Interestingly, genes that have closer distances between nSIMBA binding site to their transcription start sites (TSSs) were suppressed more significantly (Fig. 5i), indicating that the suppression of SIMBA is relatively local. In addition, the downregulated genes from Supplementary Data 2 were separated into two groups, “closed” and “open”, based on the accessibility of their promoter regions (DNase-seq data of HEK293T cells from the ENCODE project accession number “ENCFF680DCW”), and we observed more significant suppression at the closed chromatin regions, suggesting that other transcription modulators at the closed heterochromatin region may facilitate the gene suppression (Supplementary Fig. 12).

## Discussion

In this work, we developed SIMBA, an inducible BA system based on multilayer and multivalent molecular assembly involving HP1α and SunTag arrays, which can concurrently label genomic loci and manipulate local expression. The recruitment of FRB-mCherry-HP1α at the targeted loci via inducible FKBP-FRB heterodimerization can be amplified by the tandem-repeats of SunTag as well as dimerization and oligomerization of HP1α leading to biomolecular assembly formation, which can label both repetitive and non-repetitive loci with one sgRNA. The SIMBA-mediated HP1α assemblies can further recruit histone H3K9 methyltransferase SUV39H1/2 to the target sites for local gene suppression, potentially by increasing the level of suppressive H3K9me3 at the local chromatin for the formation of suppressive chromatin structure^12^. SIMBA can also be guided by endogenous transcription factors, such as NFAT1 (Fig. 4), to perturb the innate signaling and cellular functions mediated by these regulators. As such, SIMBA can be applied to track, in principle, any genomic locus in living cells, and manipulate the corresponding gene expression. Our SIMBA method should also be readily extendable to target other signaling molecules and reprogram cell fate decisions^56–58^.

A critical difficulty with conventional labeling approaches of genomic loci in single live cells is low SNR, engendered from the limited copy number of target sequences. Multiple elegant approaches have been developed to address this challenge. For example, sgRNA coupled with 16 repeats of MS2 was used for the labeling of non-repetitive regions of *MUC4* with four tiling sgRNAs^20^. Similarly, the Casilio system utilizing repeats of RNA loops at the sgRNA tail to recruit multiple probes was applied to monitor non-repetitive loci with one single kind of sgRNA^59^. To reduce the background noise, split fragments of fluorescent proteins have been fused to either RNA loop-binding domains or GCN4 scFv to form intact fluorescent proteins only when these non-fluorescent fragments are recruited to the same locus^16,22^. The LiveFISH technology has also been shown to reduce background noise via the degradation of limited amount of unbound Cy3-crRNA^60^. However, at the current stage, there is still a lack of well-established and broadly applicable method using one sgRNA to monitor non-repetitive loci in living cells. To a large extent, the weak signal from the target loci with low or no repeats, as well as the low efficiency of delivering multiple sgRNAs and the non-specific aggregation of tagged sgRNA transcripts presented major difficulties^22^. We employed the design of dCas9 carrying multiple repeats of SunTag to amplify the signals and improve the SNR of locus-specific labeling, similar to the approach using dCas9-24xSunTag to label non-repetitive regions of *MUC4* with 20 different tiling sgRNAs^26^. This amplification by SunTag tandem repeats is further enhanced by the inducible FKBP-FRB dimerization and the subsequent recruitment of HP1α, which triggers more copies of HP1α accumulation via its chromo shadow domain and ensuing multivalent polymerization leading to condensation. The resulting biomolecular assemblies at the dCas9-defined loci provides multilayer and multivalent amplification of signals, leading to efficient labeling of repetitive, as well as non-repetitive, loci with a single sgRNA. The biomolecular assemblies correctly anchored at genomic loci via dCas9 can also be more stable than the non-targeted dCas9 complexes as HP1α recruited at the loci can directly interact with neighboring DNA and histone tails to facilitate the BA stability^7^, in addition to the enhanced stability of the genomic-target-bound dCas9 complex as previously reported^60^. Efficient labeling could also be due to recruitment of SUV39H1/2, which generates more HP1α binding sites around the target locus to amplify SIMBA signal. Robust formation of FUS_N_ assemblies upon rapamycin addition was also observed (Supplementary Fig. 3d), suggesting that IDR-mediated molecular interactions and BA stability, among other factors, can be the main contributor for efficient SIMBA labeling. Due to the multiple repeats of SunTag per dCas9-nxSunTag fusion protein, to saturate the SunTag repeats, there needs to be many more copies of HP1a compared to dCas9-nxSunTag. This creates an unfavorable stoichiometry to trigger BA formation for the diffusing dCas9-nxSunTag. These factors may have contributed to the excellent contrast of locus labeling by SIMBA with a single sgRNA in a single live cell, even for non-repetitive sequences. The inducible design of SIMBA also allows the convenient identification of target loci where the specific labeling signals appear only after rapamycin treatment in the same cell. SIMBA can hence provide a robust tool for the dynamic tracking of genomic loci with high SNR in living cells.

SIMBA may regulate local gene expression by the recruitment of SUV39H1 (Fig. 5). SUV39H1 and potentially its accompanying factors can increase the H3K9me3 levels which can further recruit suppressive epigenetic and genetic modulators for local gene expression. Indeed, the inhibition of SUV39H1/2 by chaetocin treatment, H3.3K9M expression, as well as knockdown with siRNA, can significantly reduce the level of gene suppression from SIMBA (Fig. 5f–i). In addition, with 1x SunTag where biomolecular assembly did not occur, gene expression of NYAP1 was still suppressed, suggesting puncta formation may not be required for the transcriptional repression (Supplementary Fig. 10). This is consistent with a previous design in which LacI-HP1α was recruited to LacO sites and specifically suppressed associated gene expression through the tri-methylation of H3K9^12^. A similar approach (EpiGo-KRAB) was developed to target KRAB at genomic loci via dCas9 for the subsequent recruitment of SETDB1 to introduce H3K9me3. This can then lead to the recruitment of HP1α for de novo heterochromatin formation to cause gene suppression in different domains of human chromosome 19^61^. However, EpiGo-KRAB did not lead to a general gene suppression, possibly reflecting a lower level of EpiGo-KRAB comparing to that of SIMBA whose power can be amplified by the multivalent and multilayer interactions mediated by HP1α and tandem-repeats of SunTag. We cannot exclude the possibility though that SIMBA may also suppress the local transcription at a specific genomic site through the concurrent reorganization of chromatin structure. A recent report suggests that the recruitment of a large number of copies of HP1α at a target genomic region with tandem repeats can trigger large-scale heterochromatin formation and thus suppress gene expression, without the alteration of overall H3K9me3 level^42^. It is also possible that SIMBA with condensed HP1α accumulation at a local genome site may trigger the formation of suppressive BAs, which contributes to a gene suppression by occluding the genome space and physically excluding the RNA polymerase and transcriptional machinery^38,54^.

While mouse HP1α accumulation in living cells has recently been reported to share the same phase as the surrounding environment^62^, SIMBA may involve, possibly in part, liquid-liquid phase separation (LLPS) resulting from multilayer and multivalent interactions mediated by SunTag arrays and HP1α. Several lines of evidence support this notion. First, SIMBA demonstrated that their contents exchange with the surrounding environment as shown by the FRAP results (Supplementary Fig. 4e, f); Second, the disruption of multivalent interactions by 1,6-HD caused the disassembly of SIMBA (Supplementary Fig. 4a); Lastly, SIMBA underwent fusion among different puncta when guided by NFAT1 to target multiple loci (Supplementary Video 1). These observations are consistent with earlier reports that HP1α can form droplets via LLPS both in vitro and in vivo^7,8^, with neighboring co-factors (e.g., DNAs and RNAs) and weak molecular interactions in the local subcellular microenvironment potentially capable of further promoting LLPS^10,11,38^. This is also consistent with a more recently published study suggesting that the induced accumulation of many copies of HP1α at a local genome region can lead to behaviors consistent with LLPS^42^. Although HP1α was reported to promote heterochromatin formation in living cells without strong evidence of LLPS characters, the HP1α concentration in these cells was relatively low (~3 μM), much lower than the LLPS concentration requirement of ~40 μM measured by in vitro experiments^62^. Nevertheless, we do not exclude the possibility that the SIMBA assembly forms through mechanisms other than LLPS^63–65^.

Our results have suggested that the recruitment of HP1α molecules to dCas9-24xSunTag upon rapamycin treatment through FKBP-FRB dimerization can lead to a high local concentration of HP1α at the target loci, which can form biological assemblies through dimerization and oligomerization^7^. The high local concentration of HP1α may be important for the formation of these biological assemblies as dramatic decrease of labeling efficiency was observed when the fused copy number of SunTag is less than four (Supplementary Fig. 3a, b). Moreover, the interactions of HP1α molecules are critical for formation of SIMBA signals. Loss of the chromo shadow domain, which is required for HP1 dimerization and protein partner interaction, abrogated formation of BAs (Supplementary Fig. 3c). This can also be demonstrated by the fact that when HP1α was replaced by the multivalent domain FUS_N_, robust labeling can also be achieved (Supplementary Fig. 3d). In addition, HP1 oligomerization recruits additional dCas9 from the freely diffusing pool, as revealed in our experiment when dCas9 was tagged with EGFP (Supplementary Fig. 3e), resulting in even higher levels of HP1 bound to their associated SunTag and hence higher local SIMBA signal enrichment.

In addition, although the SIMBA system does not rely on endogenous HP1α for BA formation and hence does not have specific requirements for target locus selection, the applicability of SIMBA may still be limited by the genome/chromatin accessibility (as determined by dCas9 or other DNA binding proteins) or the transcriptional characteristics of the genomic locus. The recruitment of high levels of HP1α required for SIMBA, plus the potential requirement for H3K9 methylation could possibly alter the chromatin landscape of the target site and thus influence its normal spatial positioning and function in a heritable manner^12,66^. The chromatin binding property of HP1α may also cause perturbation of the target gene. For simple locus labeling without perturbation of gene expression, we have tested replacing HP1α with FUS_N_, a widely-used intrinsically disordered region without native chromatin functions, which also demonstrated robust genomic locus labeling capability (Supplementary Fig. 3d). This finding that FUS_N_ is sufficient to replace HP1α in SIMBA for locus labeling, opens up the possibility that other condensate-forming proteins could be incorporated into SIMBA, highlighting its potential modularity, particularly with condensate-forming proteins that are native to the DNA sequences of interest, which are not supposed to alter the chromatin landscape or impact spatial positioning. This notion is supported by the observation that FUS_N_ itself does not cause measurable gene expression of the target loci^67^.

**Fig. 6:**
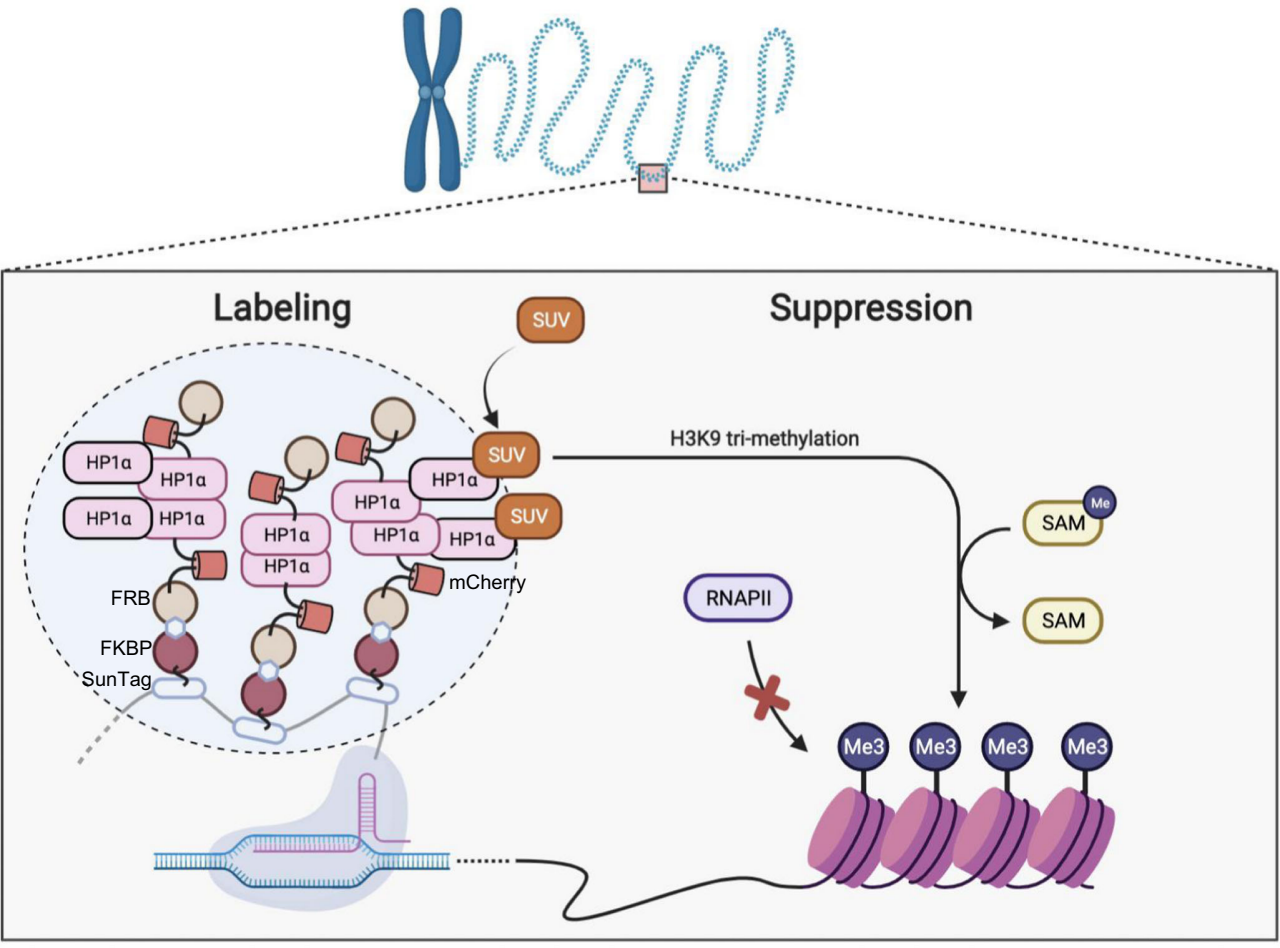
A mechanistic diagram showing the inducible SIMBA for concurrent imaging and manipulation of genomic loci in living cells. In the SIMBA system, multivalent HP1α is recruited to genomic loci via dCas9-24xSunTag (or other DNA binding domains) and chemically inducible FKBP-FRB heterodimerizer, which allows the multilayer polymerization and biomolecular assemblies at specific loci, including both repetitive and non-repetitive ones, thus labeling the genomic loci with high SNR. HP1α can recruit SUV39H1 to methylate the nearby histones, which may promote heterochromatin formation surrounding the loci, thus suppressing gene expression. It was created with BioRender.com.

In summary, we developed the platform technology SIMBA for concurrent imaging of genomic loci and manipulation of their associated gene expression in living cells, based on biomolecular assemblies and chromatin modifications (Fig. 6). With enhanced labeling signals, as well as reduced background noise, SIMBA can be applied for imaging of low- and non-repetitive loci in living cells with a single sgRNA. The interaction of SUV39H1/2 and HP1α may further induce H3K9me3 to recruit histone writers and propagate suppressive histone markers to adjacent nucleosomes, hence inhibiting the transcriptional machinery and suppressing local gene expression.

## Methods

### Plasmids

The dCas9-24xSunTag construct was derived from pHRdSV40-NLS-dCas9-24xGCN4_v4-NLS-P2A-BFP-dWPRE (Addgene #60910). Construct scFv-FKBP was generated by Gibson Assembly with scFv fragment amplified from pHR-scFv-GCN4-sfGFP-GB1-NLS-dWPRE (Addgene #60906). FRB-mCherry-HP1α was generated from previous constructs containing FRB, mCherry, and HP1α by Gibson Assembly. HP1α was amplified from human cDNA as a kind gift from Dr. Wei Wang lab at UCSD. Both scFv-FKBP and FRB-mCherry- HP1α were cloned into the pHR lentivirus backbone and expressed by the PGK promoter. N-Venus-MCP and C-Venus-PCP were constructed based on the split Venus BiFC pair from pBiFC-bJunVN155(I152L) (Addgene #27098) and pBiFC-bFosVC155 (Addgene #22013). N-Venus and C-Venus were inserted into pHAGE-EFS-MCP-3XBFP-NLS (Addgene #75384) and pHAGE-EFS-PCP-3XGFP-NLS (Addgene#75385) in between BamHI and XhoI to replace 3xBFP and 3xGFP, respectively. The gRNA constructs with MS2-PP7 loops were generated based on pLH-sgRNA1-MS2-PP7 (Addgene #75392). The guide RNA sequence for *Telomere* was 5′-tagggttagggttagggtta-3′. gRNA for *MUC4-E3* was 5′-gtggcgtgacctgtggatgctg-3′. The sequences of these two gRNAs were from a previous publication^17^. The guide RNA sequence for non-repetitive *MUC4* (*MUC4.1*) was 5′-gtaaagtagaaaaggcataaa-3′^20^. The guide RNA sequence for *IL-1B* was 5′-tgagataattctctggttca-3′^68^. For the assembly of each sgRNA in to pLH-sgRNA1-MS2-PP7 (Addgene #75392), the Golden Gate cloning method was used for replacing *CcdB* gene with each guide RNA sequence between two *BbsI* sites by following the protocol from the previous publication^19^. Briefly, annealing of sgRNA oligos was done using the following reaction mix and thermocycle: 40 μl annealing buffer (100 mM Tris-HCl pH8.0, 50 mM NaCl, 1 mM EDTA), 5 μl 100 μM Forward oligo, 5 μl 100 μM Reverse oligo. Incubate at 95 °C for 3 min, and then slowly cool down to 25 °C with ramp rate 0.5 °C/s. Then dilute 5 μl of annealed oligos to 245 μl water to make a final concentration of 200 nM (~3 ng/μl, ready for use). To clone annealed oligos into vector, 10 μl Golden Gate reaction mix was prepared and run as below: 100 ng pLH-sgRNA1-MS2-PP7, 1 μl 10 x CutSmart Buffer (B7204, New England BioLabs), 1 μl 10 mM ATP (P0756S, New England BioLabs), 0.5 μl BbsI (R3539S, New England BioLabs), 0.3 μl T7 DNA ligase (M0318S, New England BioLabs), 1 μl 200 nM Annealed oligos, add ddH_2_O up to 10 μl. Incubate at 37 °C for 15 min and transform 2.5 μl reaction mix into 25 μl *Stbl3* competent cells (C737303, Invitrogen) for further amplification to obtain single clones. TetR-24xSunTag-P2A-scFv-FKBP was generated based on TetR-EGFP plasmid from our early work^47^. The all-in-one constructs NFAT1-8xSunTag-P2A-scFv-FKBP-T2A-FRB-mCherry-HP1α and dCas9-8xSunTag-P2A-scFv-FKBP-T2A-FRB-mCherry-HP1α were based on the PiggyBac vector PB-iMYOD1-P2A-GFP-Puro. YPet/mCherry-SUV39H1ND was based on full-length SUV39H1, a kind gift from Prof. Ya-Hui Chi group at National Health Research Institute, Taiwan. Amino acid residues 1-39 were used for the SUV39H1ND domain. DNA oligo primers were synthesized at Integrated DNA Technologies (IDT Inc). PCR reactions were performed using Q5 DNA polymerase system (M0491L, New England BioLabs) following the manufacturer’s instructions. The amplified DNA fragments were purified by agarose gel electrophoresis and extracted using the Zymoclean Gel DNA Recovery Kit (D4001, Zymo Research). Gibson assembly reactions were performed using the Gibson Assembly master mix (E2611L, New England BioLabs) based on the manufacturer’s protocol. All the constructs were verified by Sanger sequencing (Genewiz).

To optimize the delivery efficiency of different components of dCas9-SIMBA, we also generated an all-in-one construct (dCas9-8xSunTag-P2A-scFv-FKBP-T2A-FRB-mCherry-HP1α), which was co-transfected with the corresponding sgRNA into HEK293T cells for locus targeting. Construct of scFv-FKBP-T2A-FRB-mCherry (HP1α deleted) was subcloned from scFv-FKBP-T2A-FRB-mCherry-HP1α. Constructs of dCas9 with different repeats of SunTag were generated based on pHRdSV40-NLS-dCas9-24xGCN4_v4-NLS-P2A-BFP-dWPRE (Addgene #60910) by Gibson Assembly. dCas9 fragments were amplified with the following primers. 4x-fwd, 5′-ggtagtgggagcaacggcagcagcg-3′; 4x-rev, 5′-ctgacccgagcctccagaaccactgcccttttttagccgag-3′; 2x-fwd, same as 4x-fwd; 2x-rev, 5′-ctgacccgagcccccacttccgctcccctttttcag-3′; 1x-fwd, same as 4x-fwd; 1x-rev, 5′-ctgacccgagcctccactgccagaacctttcttaagac-3′. The corresponding BFP fragments were amplified with the following primers. (4x) BFP-fwd, 5′-agtggttctggaggctcgggtcagcggccgcaaggtg-3′; (4x)BFP-rev, 5′-tacctcagtgatcgatccctgcagg-3′; (2x)BFP-fwd, 5′-agcggaagtgggggctcgggtcagcggccgcaaggtg-3′; (2x)BFP-rev, same as (4x)BFP-rev; (1x)BFP-fwd, 5′-tctggcagtggaggctcgggtcagcggccgcaaggtg-3′; (1x)BFP-rev, same as (4x)BFP-rev. The corresponding dCas9 and BFP fragments were inserted in dCas9-24xGCN4_v4-NLS-P2A-BFP-dWPRE vector digested with BamHI and SalI by Gibson Assembly (New England Biolabs E2611L). Constructs of HP1α truncation mutants were generated by Gibson Assembly. HP1α (delta 1-72) was cloned from scFv-FKBP-T2A-FRB-mCherry-HP1α with forward primer 5′-agcggcgggaccatgaaggagggtgaaaataataaac-3′ and reverse primer 5′-gtcacgcatgttgcaggtgggagttg-3′. HP1α (delta73-114) was cloned from scFv-FKBP-T2A-FRB-mCherry-HP1α with forward primer 5′-tctcctcttcctcatctccgggcctttc-3′ and reverse primer 5′-ctcaaagccccgcttcttatactttttcataaattcagaaattag-3′ (AA 1-72); forward primer 5′-aagtataagaagcggggctttgagagaggactggaac-3′ and reverse primer 5′-gtcacgcatgttgcaggtgggagttg-3′ (AA 115-192). HP1α (delta 115-176) was cloned from scFv-FKBP-T2A-FRB-mCherry-HP1α with forward primer 5′-tctcctcttcctcatctccgggcctttc-3′ and reverse primer 5′-atcctcaggataagcgatatcattgctctgctctctct-3′ (AA 1-114); forward primer 5′- aatgatatcgcttatcctgaggatgcggaaaacaaagag-3′ and reverse primer 5′-gtcacgcatgttgcaggtgggagttg-3′ (AA 177-192). FUS_N_ was cloned from pHR-FUSN-mCh-Cry2WT (Addgene #101223) with forward primer 5′-agcggcgggaccatggcctcaaacgattatacccaacaag-3′ and reverse primer 5′-cacgcatgttgcaggtgggagttgcaccttgcgcttcttcttgggactagttcctccacctccacggtcctgctgtc-3′; scFv-FKBP-T2A-FRB-mCherry was cloned with forward primer 5′-ctcttcctcatctccgggcctttcg-3′ and reverse primer 5′-gtttgaggccatggtcccgccgcttccccccttgtac-3′; and subcloned into parental vector digested with *EcoRI* and *NotI* by Gibson Assembly.

### Cell culture

HEK293T cells (ATCC, CRL-3216^TM^) were cultured in Dulbecco’s modified Eagle’s medium (DMEM) supplemented with 10% (vol/vol) heat-inactivated fetal calf serum (FBS), 2 mM l-glutamine, 100 units/mL penicillin, 100 units/mL streptomycin, and 1 mM sodium pyruvate in incubator at 37 °C with 5% CO_2_. All the cell culture reagents were purchased from Invitrogen. Human U2OS cells (ATCC, HTB-96^TM^) and U2OS 2-6-3 cell line (a gift from Xavier Darzacq’s lab at UC Berkeley^52^) were cultured in McCoy’s 5 A (modified) medium (Gibco, 16600082) supplemented with 10% (vol/vol) heat-inactivated fetal calf serum (FBS), 2mM L-glutamine, 100 units/mL penicillin, 100 units/mL streptomycin in incubator at 37 °C with 5% CO_2_. 96-8 HCT116 cell line with engineered TetO repeats was a gift from Huimin Zhao’s lab (UIUC)^47^ and cultured in McCoy’s 5 A (modified) medium and 10% Tet System Approved FBS (Takara, 631107).

### Plasmid transfection

Transfection was performed in 12-well plates using lipofectamine 3000 (Invitrogen), following the manufacturer’s instructions. For regular SIMBA transfections, the ratio of plasmids containing dCas9-24xSunTag and sgRNA (60 ng dCas9-24xSunTag, 60 ng scFv-FKBP, 60 ng FRB-mCherry-HP1α, 300 ng sgRNA) was adjusted to obtain minimum background signals, mainly based on the ratio of the components from CRISPRainbow system^19^. For telomere and MUC4 double labeling experiments, 60 ng dCas9-24xSunTag-P2A-BFP, 300 ng MUC4sg/Telosg-MS2-PP7, 60 ng scFv-FKBP, 60 ng FRB-mCherry-HP1α, with 8 ng MCP-N-Venus and 8 ng PCP-C-Venus were co-transfected into HEK293T cells. Cells were seeded onto glass-bottom dishes 24 hrs following transfection and imaged 36-48 hrs post transfection.

### Lentiviral vector preparation and cell line generation

Coding sequences of SIMBA components (dCas9-8xSunTag-P2A-eGFP, scFv-FKBP-T2A-FRB-mCherry-HP1α, sgRNA) were sub-cloned into a pHR lentiviral vector (Addgene #79125) to generate the corresponding lentiviral constructs. For virus preparation, HEK293T cells were seeded into 10-cm dishes one day before transfection. A total of 5 μg of pCMV-VSVG (Addgene #8454), 5 μg of pCMV delta R8.2 (Addgene #12263), and 10 μg of the corresponding SIMBA lentiviral plasmids were co-transfected into HEK293T cells by calcium phosphate transfection (Promega E1200) following the manufacturer’s protocol. Fresh medium was added at 6 hrs after transfection. Supernatant was harvested at 48 hrs post transfection, filtered through 0.45 μm, and concentrated (Takara, 631232). Aliquots of concentrated viruses were stored at –80 °C. For cell line generation, human U2OS cells were infected with the corresponding concentrated SIMBA virus diluted tenfold in DMEM and supplemented with 4 mg/ml polybrene for 24 hrs, followed by FACS sorting of positive cells on day 3 post infection. The SIMBA stable cell lines were passaged for more than five times after sorting before induced for the puncta formation upon rapamycin addition.

### Microscopy

Imaging was performed using Nikon ECLIPSE-Ti microscope equipped with a 100x, 1.45 numerical aperture (NA) oil immersion objective, a charge-coupled device (CCD) camera, a unique Perfect Focus System (PFS) that automatically corrects focus drift in real time during a prolonged period of time-lapse imaging at 37 °C with 5% CO_2_. A 495DF10 excitation filter, a 510DRLP dichroic mirror, and 535DF30 emission filter were used for Venus or YPet imaging, and a 580DF20 excitation filter, a 595 DRLP dichroic mirror, and a 630DF20 emission filter were used for mCherry imaging. Time-lapse fluorescence images were acquired and analyzed by MetaMorph (version 7.8.6.0, Molecular Devices, Sunnyvale, CA) or ImageJ (2.3.0). Z-stack images were acquired at 0.5 μm step size and maximum z-projection of images was used to generate 2D-projected images. Cell culture media were changed into live cell imaging solution without phenol red (Thermo Fisher, A1896701) before imaging under microscopy.

### Signal-to-noise ratio analysis

Specific SIMBA-labeled puncta were analyzed based on signal-to-noise ratio (SNR) following reported methods for SNR analysis^25,69,70^. All the fluorescence intensity measurements were quantified by Metafluor 7.10.4.450 software (Molecular Devices, Sunnyvale, CA) and ImageJ (2.3.0). Briefly, SIMBA puncta were manually segmented and circled to quantify their average intensity. Background signals were quantified from regions in the nucleus outside of puncta and nucleolus to take the average and obtain the mean background intensity and its standard deviation. SNR was calculated by dividing the corrected SIMBA puncta signal (mean SIMBA puncta intensity minus mean background intensity) by the standard deviation of the background signal. The value of SNR = 10 was used for specific SIMBA punctum determination upon rapamycin induction. All the plots were graphed in Graphpad Prism version 8 (San Diego, CA).

### Fluorescence Microscopy and data analysis of HP1α assemblies

HEK293T cells (70-80% confluent) were transfected with SIMBA or its variants as specified in each experiment using lipofectamine 3000 (Thermo Fisher) following the manufacturer’s protocol. At 24 hrs after transfection, cells were seeded onto glass-bottom dishes coated with fibronectin (F1141 Sigma-Aldrich), and rapamycin (final 100 nM) was added 2 hrs before imaging (36–48 hrs after transfection) to induce HP1α assembly formation. To analyze the effect of SunTag repeat number in SIMBA on HP1α assemblies, HEK293T cells were transfected with SIMBA system with dCas9 carrying different repeats of SunTag. The data were acquired under the same microscope imaging settings and conditions. HP1α assemblies in each condition (24x, 4x, 2x, 1x) were compared based on the normalized fluorescence value of each punctum which was calculated as punctum mean fluorescence intensity (corrected against background intensity) multiplied by each punctum size (pixel area). Data normalization was performed in the same way for all the 4 groups.

### RT-qPCR

For *HSP70* RT-qPCR, 96-8 HCT116 cells were transfected with 100 ng TetR-24xSunTag-P2A-scFv-FKBP and 100 ng FRB-mCherry-HP1α for one well in 12-well plate with lipofectamine 3000. At 36–48 hrs post transfection, cells were pre-treated with 100 nM rapamycin or DMSO for 1 hr, and then incubated at 42 °C water bath for 30 min of heat shock stimulation before putting back to cell culture incubators for another 6 hrs incubation. For *NYAP1*, *LRRC4B*, and *NGFR* RT-qPCR, HEK293T cells were transfected with 100 ng dCas9-8xSunTag-P2A-scFv-FKBP-T2A-FRB-mCherry-HP1α and 250 ng corresponding sgRNAs. mCherry positive cells were sorted at 24 hrs post transfection (SONY SH800), and reseeded in a 24-well plate. Sorted mCherry-positive cells were treated with 100 nM rapamycin for 6 hr before harvesting the cells for RNA extraction. For SUV39H1 suppression with chaetocin, HEK293T cells were transfected with SIMBA constructs as described above, the sorted mCherry-positive cells were treated with 500 nM chaetocin (C9492, Sigma-Aldrich) for 6 hrs, followed by 100 nM rapamycin for 6 hrs before harvesting cells for RNA extraction. For SUV39H1 suppression with H3.3 K9M, HEK293T cells were co-transfected with pCBI-mCherry-H3.3K9M and SIMBA. For SUV39H1/2 suppression with siRNA, the HEK293T cells were transfected with 20 nM hs.Ri.SUV39H1.13.1 and 20 nM hs.Ri.SUV39H2.13.1 (IDT) with Lipofectamine 3000 following the manufacturer’s protocol. Total RNAs were extracted using RNeasy Mini Kit (QIAGEN, 74104) and converted into cDNA using SuperScript IV Reverse Transcriptase (Invitrogen, 18090050). Quantitative PCR was performed by using SYBR Green Mix (BioRad, 1725125) with qPCR primers from IDT. The qPCR primers for *HSPA1A* gene were: Forward 5′- agctggagcaggtgtgtaac-3′ and Reverse 5′-cagcaatcttggaaaggccc-3′. The qPCR primers for *NYAP1*, *LRRC4B*, and *NGFR*, were: *NYAP1*-f: 5′-ccagcaccaagctcagcatgg-3′, *NYAP1*-r: 5′-agacagagagctgggtgttagg-3′; *LRRC4B*-f: 5′-acctgcaagagaacggcatcca-3′, *LRRC4B*-r: 5′-tcgatcttgcgcaccaggttct-3′, *NGFR*-f: 5′-cctcatccctgtctattgctcc-3′, *NGFR*-r: 5′- gttggctccttgcttgttctgc-3′. Samples were run on a BioRad CRX384 Touch Real-time PCR machine. Three replicates were examined for all groups. Gene expression was quantified using the 2^−ΔΔCT^ method using beta-actin (*ACTB*) as an internal control.

### Cell sorting

For FACS experiments, cells were sorted on a SONY SH800S cell sorter equipped with lasers of 488 nm, 561 nm, and 638 nm, using sorting chips of nozel size 100 μm (LE-C3210, SONY). Sorted cells were collected in culture medium of 4 °C during the sorting process and seeded to cell culture dishes immediately after sorting following culture conditions described above. The gating strategy was shown in Supplementary Fig. 13. To investigate NFAT1 associated transcriptomic changes upon SIMBA formation at the local genome sites, HEK293T cells used for RNA-seq were transfected with 100 ng all-in-one constructs NFAT1-8xSunTag-P2A-scFv-FKBP-T2A-FRB-mCherry-HP1α or NFAT1-8xSunTag-P2A-scFv-FKBP-T2A-FRB-mCherry. 24 hrs post transfection, cells with medium fluorescence intensity were sorted out by SONY SH800. The sorted cells were continuously cultured for overnight before pre-treated with 30 μM ATP and 100 nM AP21967 (rapalog) for 1 hr, followed by another 6 hrs incubation before harvesting total RNAs by using RNeasy Mini Kit (QIAGEN, 74104).

### RNA-sequencing

HEK293T cells used for RNA-seq were transfected with 100 ng all-in-one constructs NFAT1-8xSunTag-P2A-scFv-FKBP-T2A-FRB-mCherry-HP1α or NFAT1-8xSunTag-P2A-scFv-FKBP-T2A-FRB-mCherry. The cells were treated and sorted as described above to harvest the total RNAs. The RNA samples were sent to the Genomics Center at UC San Diego Institute for Genomic Medicine (IGM) for sequencing. Briefly, RNA sample QC was performed using Agilent TapeStation system. RNA sequencing library was prepared using Illumina Stranded mRNA Prep, and sequencing performed on Illumina NovaSeq 6000 at IGM with 25 million reads per sample. Raw RNA-seq reads were first preprocessed using Ktrim v1.3.0^71^ to remove sequencing adapters and low-quality cycles; PCR duplicates (i.e., reads with identical sequences) were then removed using in-house programs and the remaining reads were aligned to the human genome (build GRCh38/hg38) using STAR v2.7.9a software^72^; expression quantifications were performed using featureCounts v2.0.3 software^73^ against ENSEMBL gene annotation v101^74^; differential expression analysis was performed using DESeq2 v1.26.0 software^75^; genes with an expression change larger than 2-fold and adjusted p-value smaller than 0.01 were considered as differentially expressed genes. For each gene, promoter region was defined as 2 kb around its Transcription Start Site (TSS). The predicted binding loci of NFAT1 were obtained from JAPSAR database^76^.

### Fluorescence recovery after photobleaching

FRAP was performed using a Nikon Eclipse Ti2 microscope equipped with two Photometrics Prime 95B cameras, a Yokogawa CSU-W1 SoRa confocal scanner unit, Nano-Drive positioning stage, SOLA light engine, Apo TIRF 100x oil DIC N3 (NA = 1.49, WD = 120 µm) objective, and 405 nm and 561 nm lasers. Identified puncta were imaged every 3 s for 30 s before photobleaching with 405 nm laser at 100% power, and further observed every 3 sec for 3 min. Data acquisition was done using NIS Elements AR 5.21.02 software. For quantification of FRAP, the fluorescence intensity values of photobleached puncta were first corrected against those of an unbleached puncta (control). The corrected intensities of different time points were then normalized to the basal level before bleaching to show the recovery dynamics. The FRAP curve was fitted with single-exponential model^77^ to estimate the recovery rate (Prism 8.0).

### Statistics and reproducibility

All the experiments were repeated independently with similar results in at least three biological replicates and successful. No statistical method was used to predetermine sample size. Sample sizes for experiments were determined based on similar published studies to provide sufficient statistical power for data analysis. No data were excluded from the analyses. The experiments were not randomized. The Investigators were not blinded to allocation during experiments and outcome assessment. The sample size, statistical significance value and error bar graphs were listed in figure legends.

### Reporting summary

Further information on research design is available in the Nature Portfolio Reporting Summary linked to this article.

## Supplementary information

Supplementary Information

Description of Supplementary Additional Files

Supplementary Data 1

Supplementary Data 2

supplementary Video 1

Reporting Summary

## Data Availability

Full data is available in the main text and supplementary materials. RNA-seq datasets are available at NCBI SRA with accession number “PRJNA905219”. The HEK293T DNase-seq data which was used in this work is available from the ENCODE project under accession number “ENCFF680DCW”. Source data are provided with this paper.

## Source data

Source Data
