# Supplementary material for: Engineering inducible biomolecular assemblies for genome imaging and manipulation in living cells
Source: Nat Commun. 2022 Dec 24;13:7933. doi: 10.1038/s41467-022-35504-x (PMC9789998; doi:10.1038/s41467-022-35504-x)
Supplement: Supplementary file 1 — Supplementary Information [file 41467_2022_35504_MOESM1_ESM.pdf]

SUPPLEMENTARY INFORMATION

Engineering Inducible Biomolecular Assemblies for Genome Imaging and Manipulation in Living Cells

Qin Peng[2#*], Ziliang Huang[1#], Kun Sun[3], Yahan Liu[1], Chi Woo Yoon[1], Reed E. S. Harrison[1], Danielle L. Schmitt[4], Linshan Zhu[1], Yiqian Wu[1], Ipek Tasan[5], Huimin Zhao[5,6], Jin Zhang[4], Sheng Zhong[1], Shu Chien[1,7], Yingxiao Wang[1*]

[1] Department of Bioengineering, Institute of Engineering in Medicine, University of California, San Diego, 9500 Gilman Drive, La Jolla, CA, 92093-0435, USA

[2] Institute of Systems and Physical Biology, Shenzhen Bay Laboratory, Shenzhen, 518132, P.R. China

[3] Institute of Cancer Research, Shenzhen Bay Laboratory, Shenzhen, 518132, P.R. China

[4] Department of Pharmacology, University of California, San Diego, 9500 Gilman Drive, La Jolla, CA, 92093-0435, USA

[5] Department of Biochemistry, University of Illinois at Urbana-Champaign, Urbana, IL, USA

[6] Department of Chemical and Biomolecular Engineering, University of Illinois, Urbana-Champaign, Urbana, IL, USA

[7] Department of Medicine, University of California, San Diego, 9500 Gilman Drive, La Jolla, CA, 92093-0435, USA

# These authors contributed equally.

* Corresponding authors: Yingxiao Wang, yiw015@eng.ucsd.edu;

Qin Peng, pengqin@szbl.ac.cn

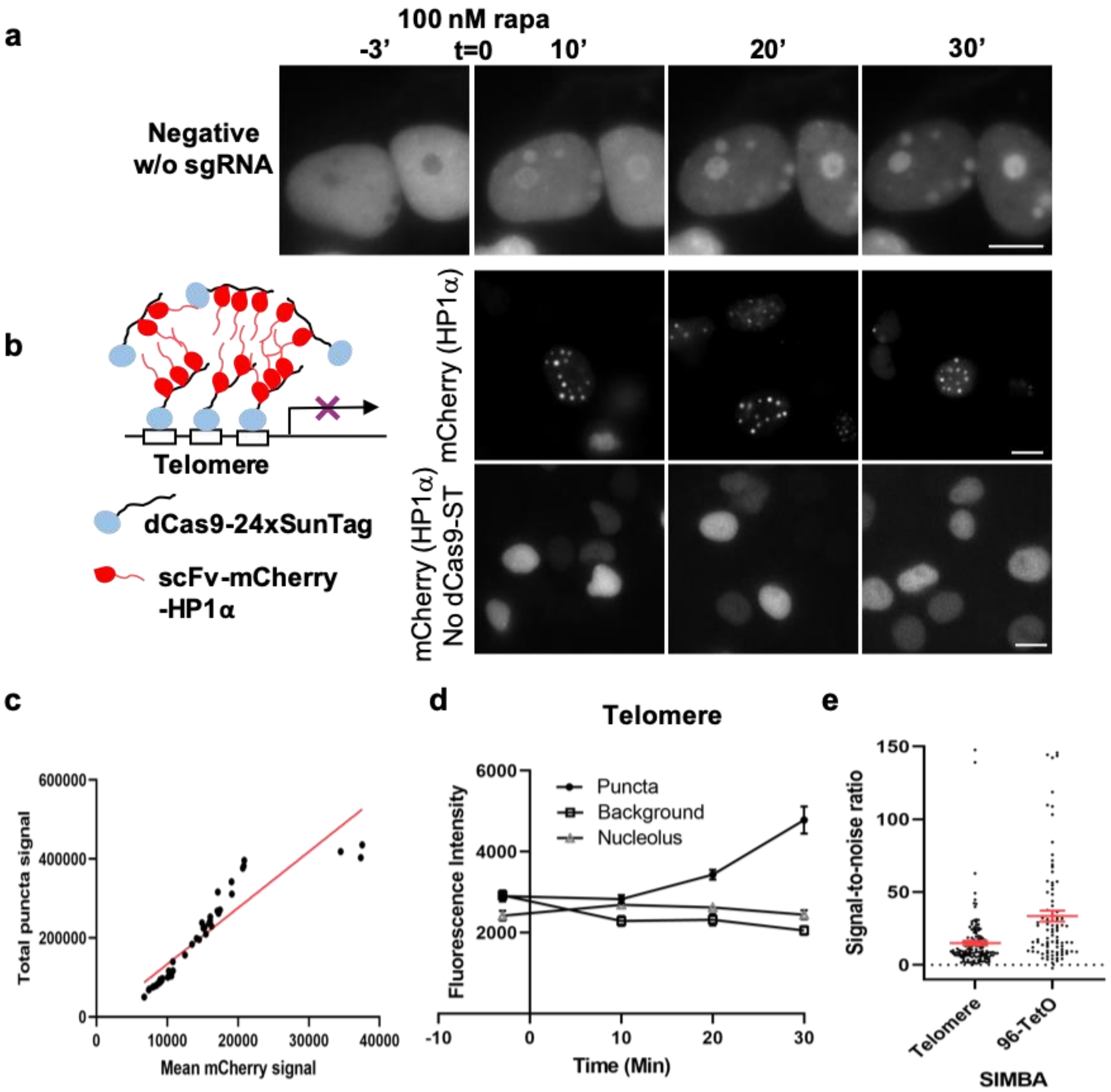

Supplementary Fig. 1. **Characterizations of SIMBA system.** (**a)** The negative control group of SIMBA without sgRNA shows no locus-specific labeling. Scale bar, 5 µm. (**b)** A SunTag-HP1α system was examined where scFv was directly fused to HP1α without the mediation of FKBP-FRB dimerization. Biomolecular assemblies can still form. Scale bar, 10 µm. **(c)** The correlation between mean mCherry signal and total puncta signal of SIMBA assembly was assessed. The total puncta intensity serves as a function of overall scFv-mCherry-HP1α concentration. The results showed that higher mCherry intensities are corresponding to higher puncta signal (*n* = 44 nuclei). **(d)** The fluorescence intensity changes in Fig. 1b were plotted on the puncta, nucleolus, and the other background nuclear region outside of puncta and nucleolus before and after rapamycin. While the mCherry intensity increased drastically in puncta, no progressive increase in nucleolar accumulation and reduced nuclear background of mCherry were observed. *n* = 6, 3, 3 selected regions in the nucleus. Error bars, mean ± SD. **(e)** Labeling efficiency of Telomere and 96-TetO

was determined by signal-to-noise ratio, n = 138, 89 puncta from more than 40 cells. Error bars, mean ± SD. Source data are provided as a Source Data file.

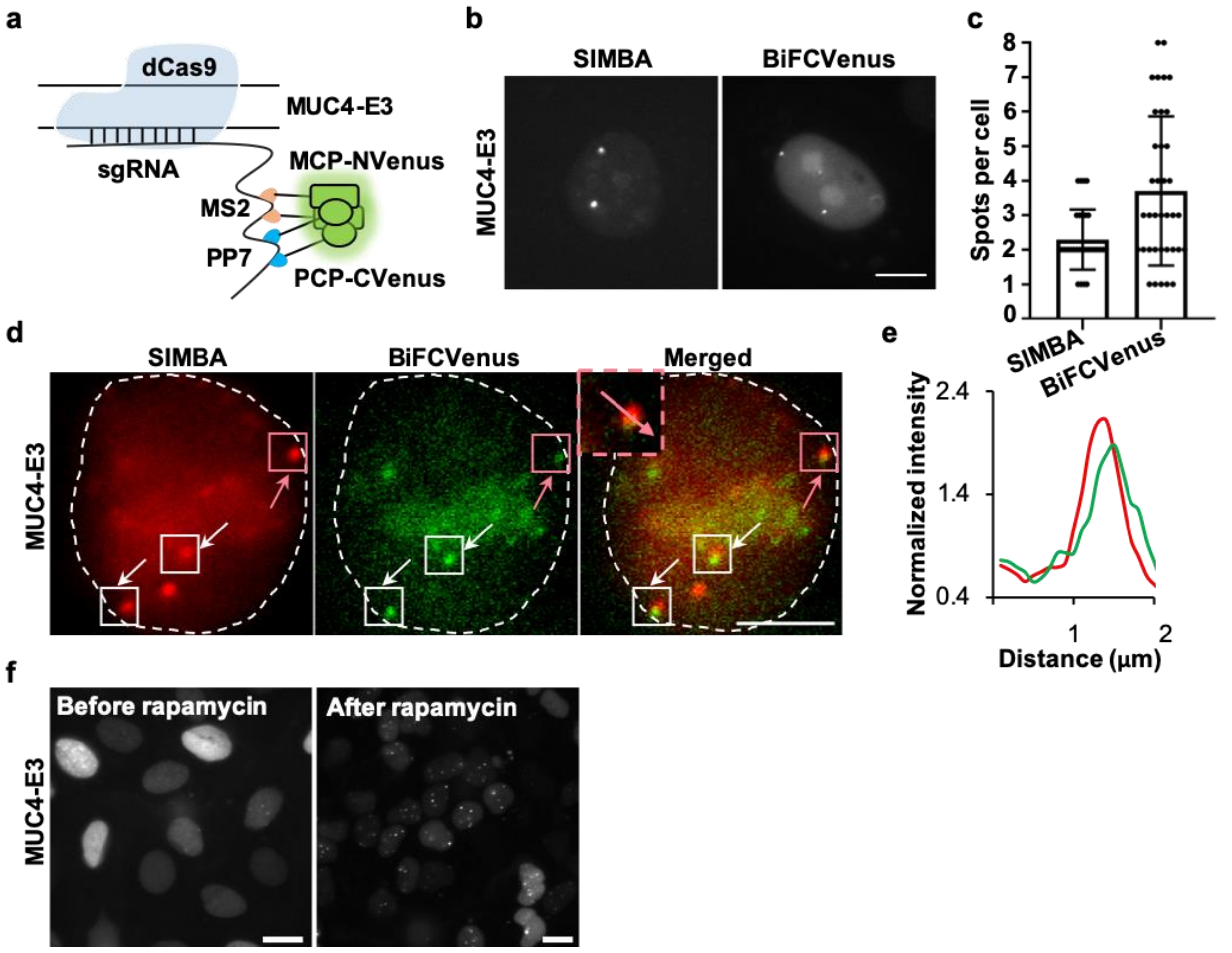

Supplementary Fig. 2. **Comparison of *MUC4-E3* locus labeling by SIMBA and BiFC. (a)** Schematic drawing of the BiFC system. (**b**) *MUC4-E3* sgRNA containing one copy of MS2-PP7 loops was co-transfected with dCas9, MCP-N-Venus, and PCP-C-Venus in HEK293T cells. Representative images show that the SIMBA labeled *MUC4-E3* locus has better contrast than that of BiFC. Scale bar, 5 μm. **(c)** Bar graph showing 2-3 MUC4 locus spots per cell in average detected by SIMBA (n = 21 cells) and BiFC (n = 25 cells). Error bars, mean ± SD. **(d)** Double labeling of *MUC4-E3* locus by applying BiFC on the sgRNA side and SIMBA on the dCas9 side. Representative images showing the labeled *MUC4-E3* locus by BiFC and SIMBA upon rapamycin treatment. BiFC channel is not labeling all foci shown in the SIMBA channel, possibly due to the limited copies at the target loci and less efficiency of BiFC. Squares and arrows highlight BAs shown in both channels. Scale bar, 5 μm. (**e**) Normalized intensities from SIMBA (red) and BiFC Venus (green) across the puncta highlighted in red squares in (**d**). **(f)** Stable human U2OS cell line engineered with SIMBA system. U2OS cells were transduced with lentiviral vectors to express dCas9-8xSunTag, scFv-FKBP-T2A-FRB-mCherry-HP1α, and sgRNA targeting *MUC4-E3*. The SIMBA stable cell lines that have been passaged for more than 5 times after cell sorting, the puncta

can still be induced upon rapamycin addition. Left, before rapamycin induction. Right, after rapamycin induction. Scale bar, 20 μm. Source data are provided as a Source Data file.

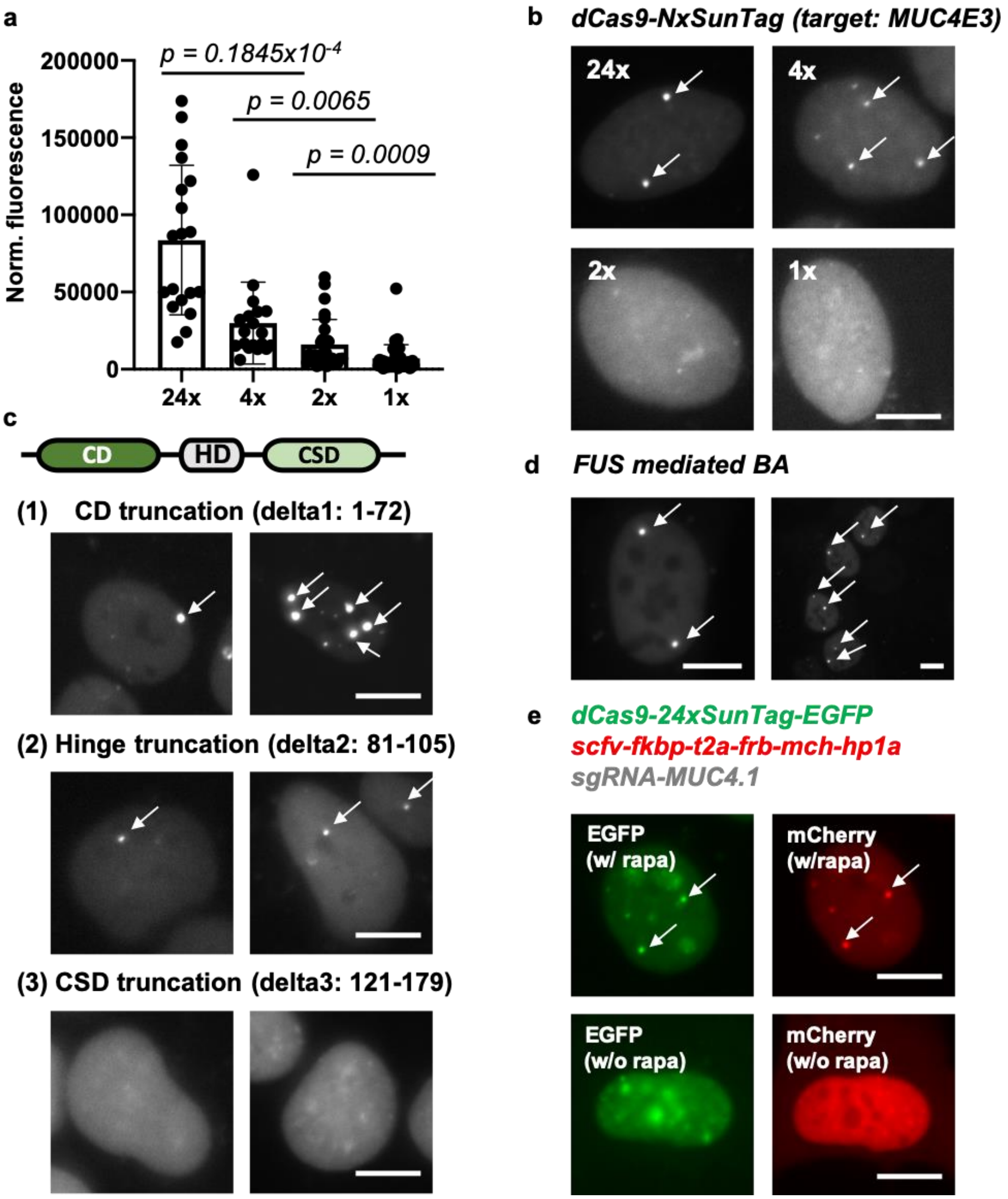

Supplementary Fig. 3. Evaluating the key factors that is critical for the SIMBA system. (a) Effect of dCas9-fused SunTag repeat number on HP1α assemblies. HP1α assemblies in each condition were compared based on the normalized fluorescence value of each punctum which was calculated as punctum mean fluorescence intensity (corrected against background intensity) multiplied by each punctum size (pixel area). The corresponding SNR for each group was calculated and included in Supplementary Data 1. The data were acquired under the same conditions, and data normalization was performed in the same way for all the 4 groups. n = 19, 19, 27, 40 nuclei for 24x, 4x, 2x, and 1x, respectively. One-way ANOVA was applied for statistical

analysis followed by Holm-Sidak's multiple comparison test (p = 0.1845x10^{-4}, p = 0.0065, p = 0.0009, respectively, as indicated in the figure). Error bars, mean ± SD. **(b)** Representative images of HP1α assemblies with different numbers of dCas9-fused SunTag repeats. Scale bar, 10 μm. **(c)** Effects of different domain truncations of HP1α on assembly formation. CD, chromodomain; HD, hinge domain; CSD, chromo shadow domain. U2OS cells were transfected with dCas9-24xSunTag, scFv-FKBP-2A-FRB-mCherry-HP1α (with the indicated truncation), and *MUC4-E3* sgRNA. Scale bars, 10 μm. **(d)** U2OS cells transfected with dCas9-24xSunTag, scFv-FKBP-T2A-FRB-mCherry-FUSN, and *MUC4-E3* sgRNA after rapamycin induction. Scale bars, 10 μm. **(e)** Representative images of U2OS cells transfected with dCas9-24xSunTag-EGFP, scFv-FKBP-T2A-FRB-mCherry-HP1α, and sgRNA *MUC4.1* with or without rapamycin induction for 3hr. Scale bars, 10 μm. Source data are provided as a Source Data file.

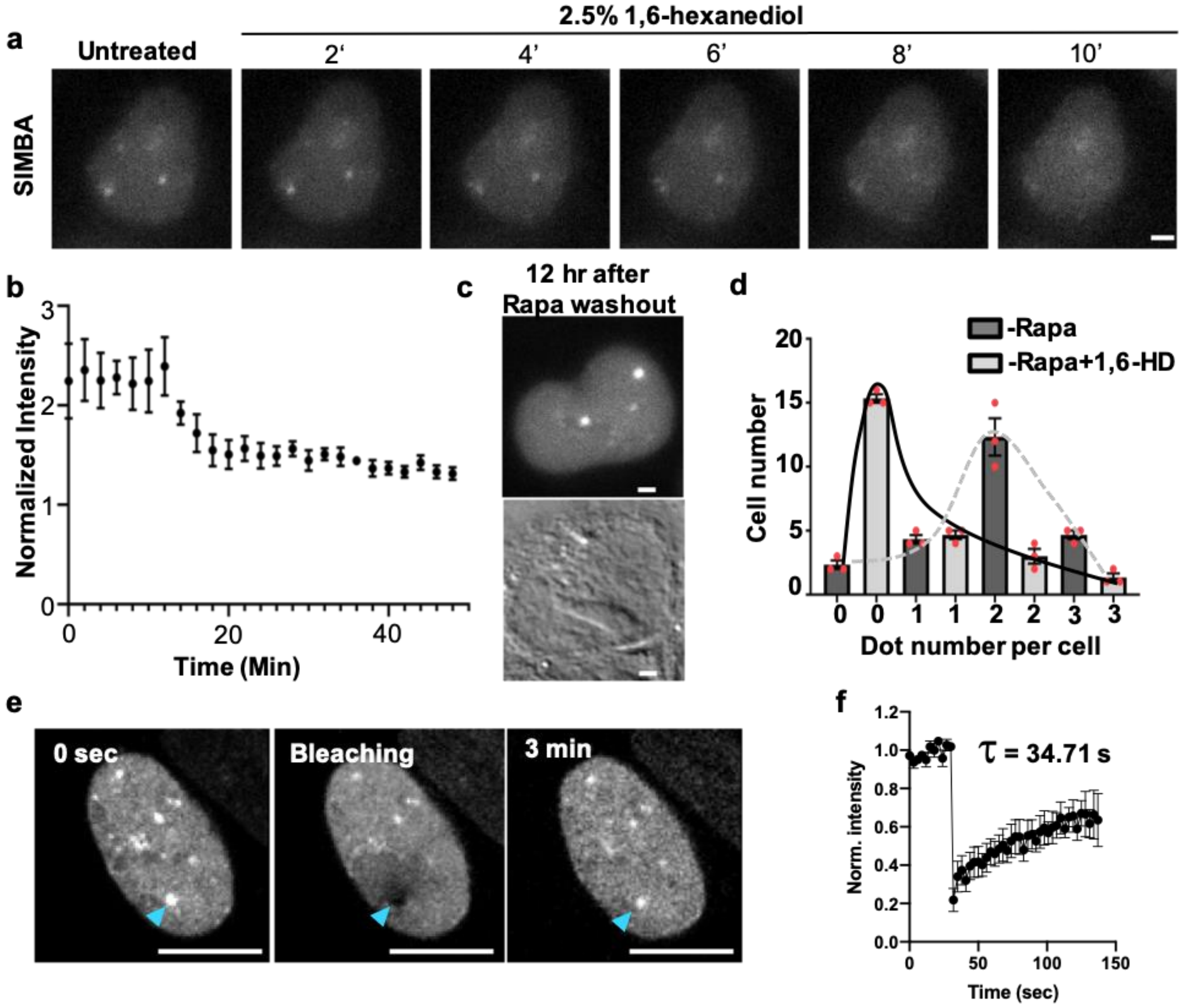

Supplementary Fig. 4. **Characterization of SIMBA stability and dynamics. (a)** Time-lapse images showing the gradual disappearance of SIMBA puncta labeling the *MUC4-E3* locus, upon 2.5% 1,6-hexanediol treatment (HD). A representative cell was shown that the assembly dissociation occurs within 10 min upon 2.5% 1,6-HD in HEK293T cells. Scale bar, 1 μm. **(b)**

Normalized intensity represents the ratio of the fluorescence intensities of the nucleus within and outside of the SIMBA puncta, before and after 2.5% 1,6-hexanediol treatment. *n* = 9 puncta. Error bars, mean ± SEM. **(c)** The images show, in the absence of 1,6-hexanediol, the remaining SIMBA puncta at the *MUC4-E3* locus after rapamycin washout for 12 hr. Scale bar, 1 μm. **(d)** The distribution of SIMBA punctum numbers in the rapamycin washout only group (-Rapa) and that with an additional 1,6-hexanediol treatment (-Rapa+1,6-HD). The puncta number was counted 12 hr post rapamycin washout and 50 min upon 1,6-HD addition of three independent experiments (*n* = 3). Error bars, mean ± SEM. (**e**, **f)** FRAP of SIMBA dots. Representative images (**e**) and time course (**f**) of the normalized fluorescence intensity of SIMBA dots before and after photobleaching. Recovery curve was fit with single-exponential model as previously reported (PMID: 26406374), with an estimated recovery rate (τ) of 34.71 sec. *n* = 9 individual FRAP events, Error bars, mean ± SEM. Scale bar, 5 μm. Source data are provided as a Source Data file.

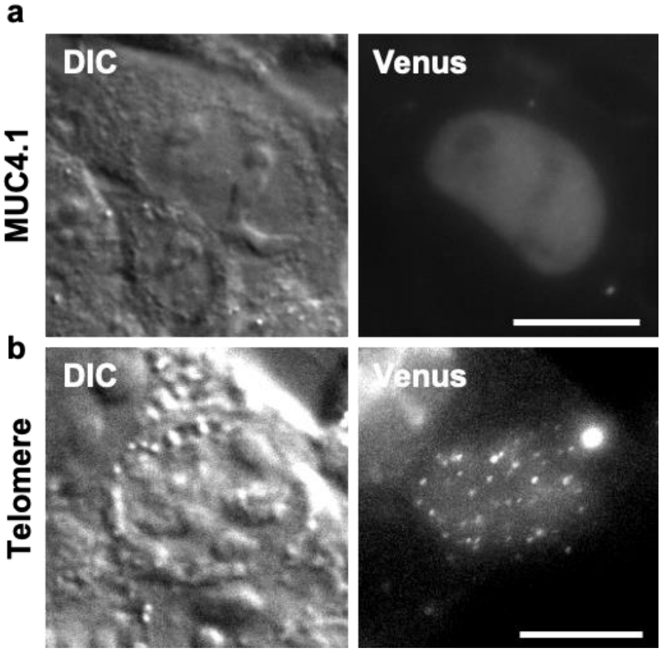

Supplementary Fig. 5. Locus labeling using sgRNA with 4xMS2-PP7 loops. HEK293T cells were transfected with dCas9, MCP-VenusN155(I152L), PCP-VenusC155, and sgRNA-*MUC4.1*-4xMS2-PP7 (**a**) or sgRNA-*Telomere*-4xMS2-PP7 (**b**) following the procedures as described in **Methods**. Imaging was performed at 48 hr post transfection. Scale bars, 10 μm.

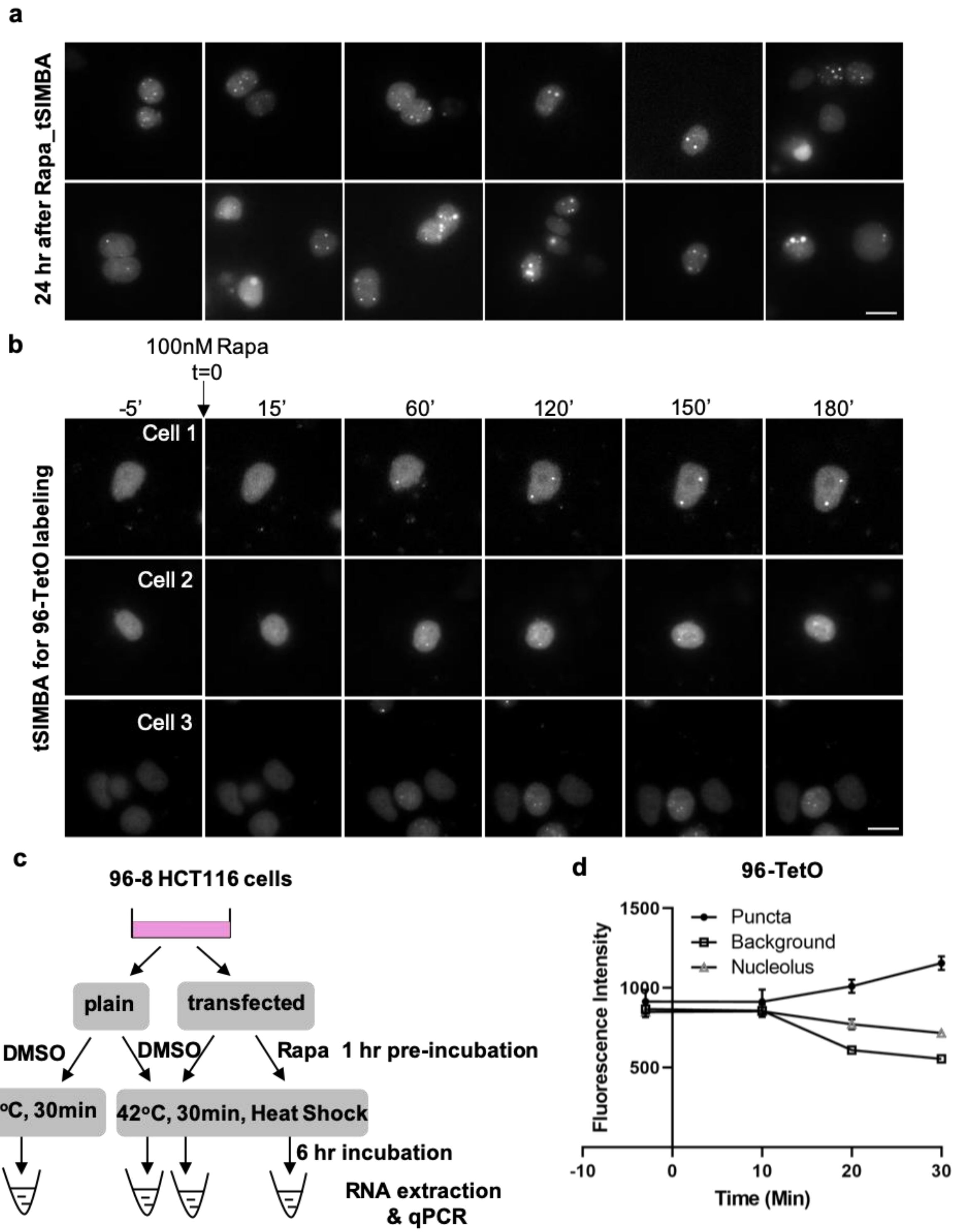

Supplementary Fig. 6. The gene suppression efficiency of tSIMBA. (a) Images of tSIMBA from multiple cells after 24 hr induction by rapamycin to show the efficiency of the method and the signal strength. tSIMBA system utilized TetR-24xSunTag for the targeting of engineered *HSP70* locus with 96-TetO inserted in HCT116 cells. No sgRNA was used in this experiment. Scale bar, 10 μm. **(b)** Another set of time-lapse images over 3 hours showed the dynamic formation

of HP1α condensates upon rapamycin stimulation in 96-8 HCT 116 cells. Scale bar, 10 μm. **(c)** The experimental design and steps of assessing the effect of tSIMBA on local gene expression. **(d)** The fluorescence intensity changes in Fig. 3b were plotted on the puncta, nucleolus, and the other background nuclear region outside of puncta and nucleolus before and after rapamycin. The mCherry intensity increased drastically in puncta. No progressive increase in nucleolar accumulation and reduced nuclear background signals of mCherry were observed. *n* =3, 5, 3 selected regions in the nucleus. Error bars, mean ± SD. Source data are provided as a Source Data file.

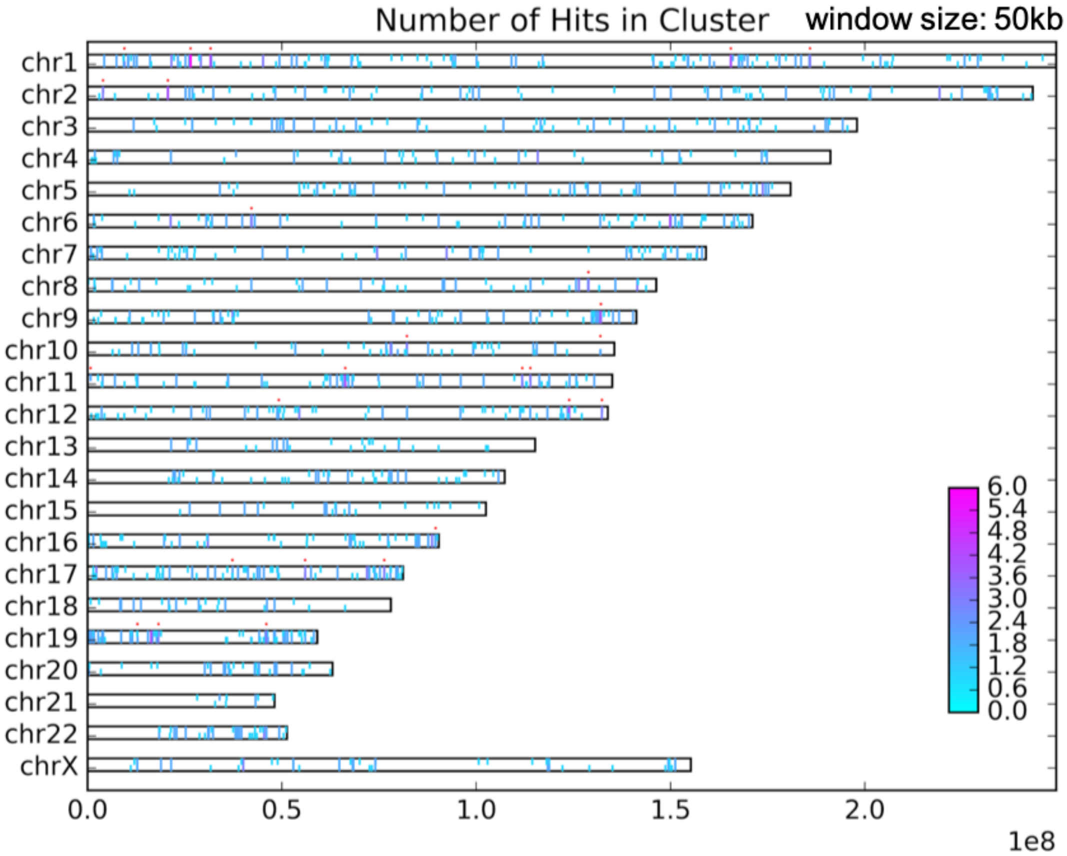

Supplementary Fig. 7. The distribution of NFAT1 binding sites in the whole genome of HEK293T cells. The displayed range of the color bar represents the number of hits in cluster. The window size is 50 kb.

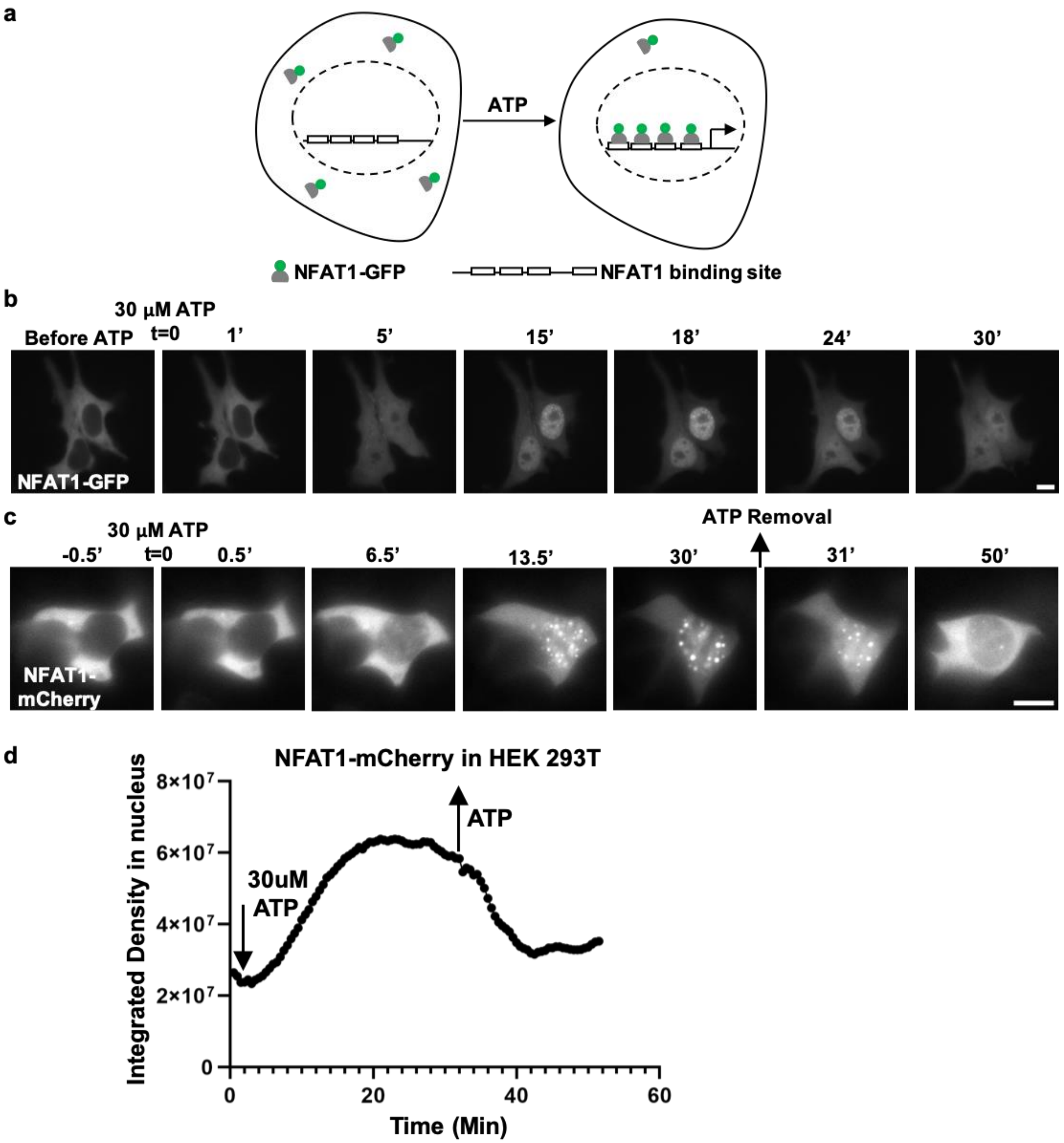

Supplementary Fig. 8. NFAT1-GFP targets and labels NFAT1 binding sites in the genome. (a) Schematic drawing showing the translocation of NFAT1-GFP into the nucleus to bind and label genome sites upon ATP stimulation. **(b)** Time-lapse images showing the genome sites in the nucleus labeled by NFAT1-GFP in HEK293T cells upon 30 μM ATP treatment. Scale bar, 5 μm. **(c, d)** Time-lapse images (**c**) and quantified integrated density (**d**) of the NFAT1 puncta upon addition and after removal of ATP. The nuclear NFAT1 aggregates without SIMBA start to reduce and NFAT1 proteins translocate back to cytosol at ~30 min after ATP stimulation, and most

nuclear NFAT1 proteins exit nucleus within 10 mins upon the removal of ATP. Scale bar, 5 μm. Source data are provided as a Source Data file.

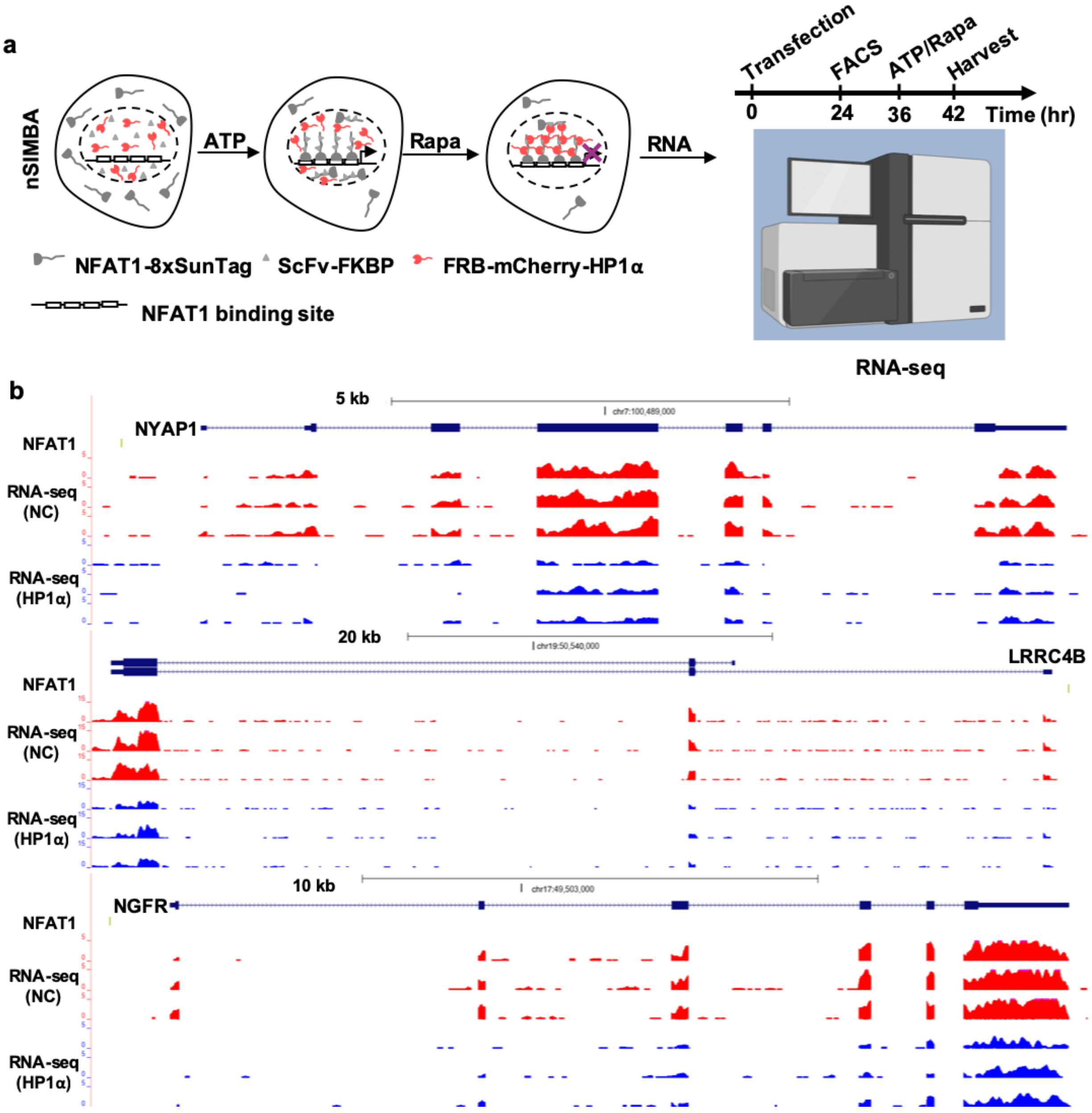

Supplementary Fig. 9. Analysis of the NFAT1 SIMBA associated transcriptome. (a) Flowchart of the design and timeline of the RNA-seq experiment. **(b)** Snapshots of *NYAP1* (top panel), *LRRC4B* (intermediate panel), and *NGFR* (bottom panel) genes. For each gene, genomic coordination (reference genome: hg38), RefSeq annotation, predicted NFAT1 binding site in JASPAR database, and normalized RNA-seq signals in negative control without HP1α (NC) and HP1α groups (each has 3 replicates) are shown.

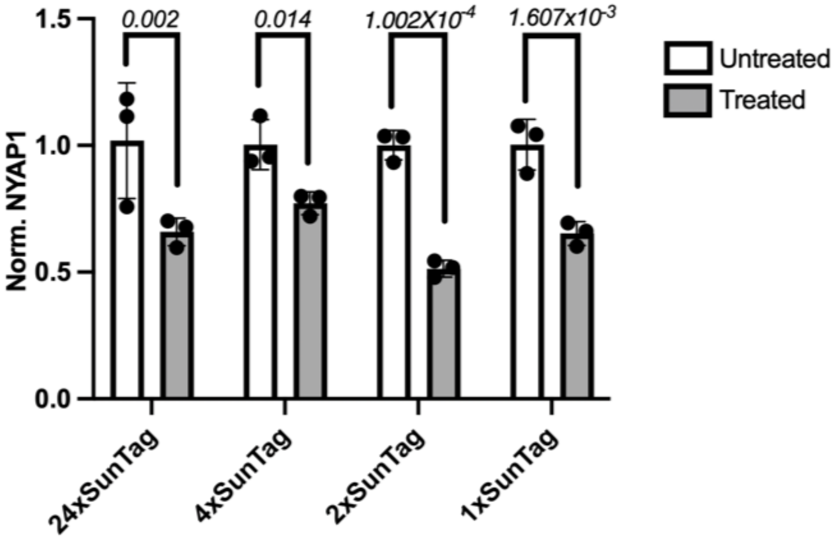

Supplementary Fig. 10. The suppression mechanism of SIMBA system. Gene suppression effect of SIMBA with different repeat number of SunTag fused with dCas9. *NYAP1* was used as target for SIMBA; transfected cells were subjected to the treatment with (treated) or without (untreated) 100 nM rapamycin analog for 6 hrs at 36 hrs post transfection. Transcript levels were normalized to that of samples without rapamycin treatment in each case. $n = 3$ biologically independent experiments, unpaired two-sided t test with Holm-Sidak's multiple comparison test; adjusted p values for multiple t-tests are indicated ($p = 0.002$, $p = 0.014$, $p = 1.002 \times 10^{-4}$, $p = 1.607 \times 10^{-3}$, respectively, as indicated in the figure). Error bars, mean ± SD. Source data are provided as a Source Data file.

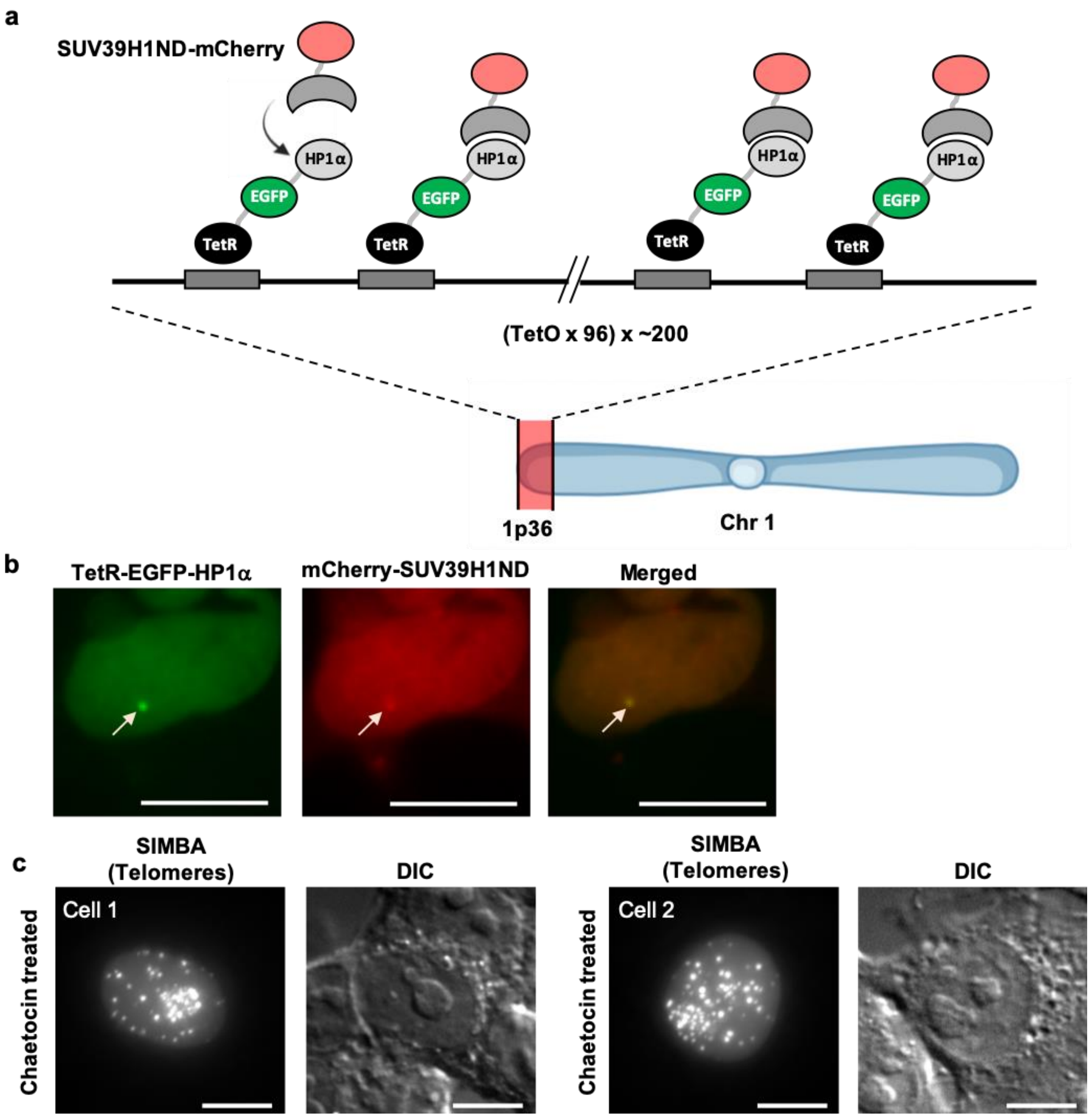

Supplementary Fig. 11. tSIMBA can recruit SUV39H1ND to the target genome site. (a) The diagram drawing depicts the expected recruitment of mCherry-SUV39H1ND by TetR-EGFP-HP1α targeting the engineered TetO site in the genome of U2OS 2-6-3 cell lines. It was created with BioRender.com. **(b)** Live cell imaging showed the accumulated signals of EGFP and mCherry at a local genome site via TetR-EGFP-HP1α targeted to the TetO repeats on Chromosome 1 for the recruitment of SUV39H1ND-mCherry. Scale bars, 5 μm **(c)** Condensate formation was not affected by chaetocin treatment (500 nM chaetocin for 6 hr) as used in Fig. 5f. The *telomere*-targeting sgRNA was used in this experiment. Scale bars, 10 μm.

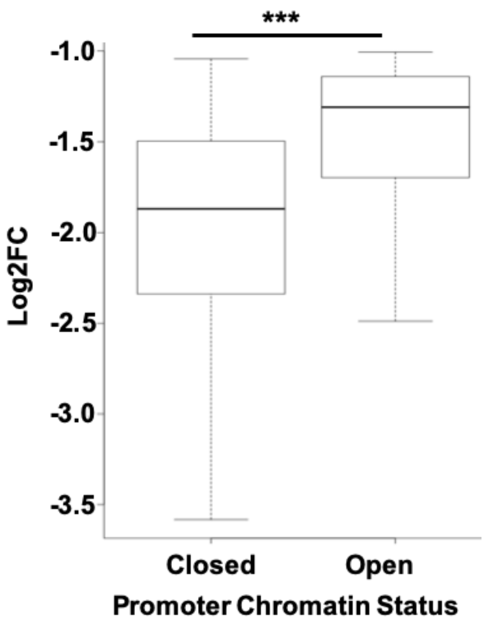

Supplementary Fig. 12. The correlation between fold change of downregulated genes and promoter chromatin status in nSIMBA system. The down-regulated genes were separated into 2 groups based on the openness (determined using Dnase-seq data) of their promoter and compared the fold-changes between these 2 groups. Processed peaks for HEK293T line were downloaded from ENCODE project (accession number ENCFF680DCW). These are standard boxplots, which contains quantiles, maximum and minimum, and no error bars. n = 185 and 164 genes, respectively. *** Mann-Whitney U test two-sided p =8.4x10^{-16}.

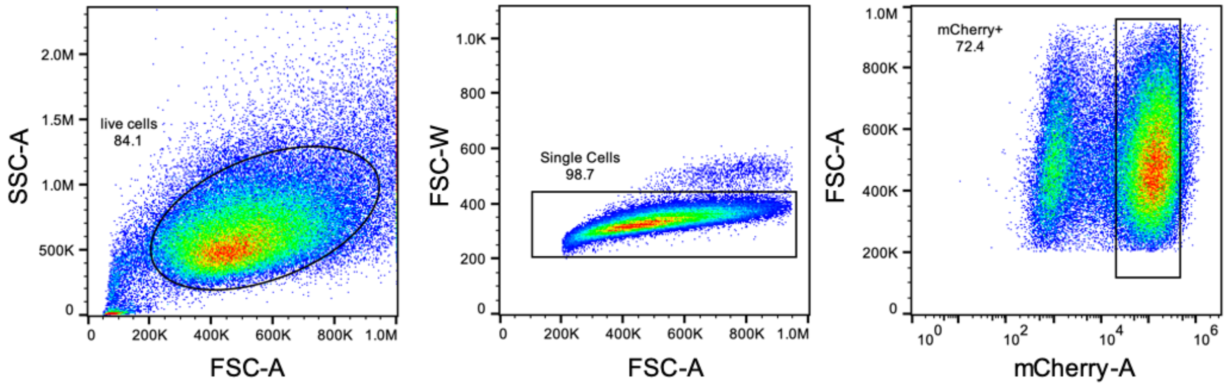

Supplementary Fig. 13. Gating strategy for FACS cell sorting. Live cells were first gated based on FSC-A/SSC-A as shown in left panel; single cells were then gated based on FSC-A/FSC-W as shown in the middle panel; transfected cells (mCherry+) were gated based on mCherry-A/FSC-A as shown in the right panel.
