# Supplementary material for: Engineering inducible biomolecular assemblies for genome imaging and manipulation in living cells
Source: Nat Commun. 2022 Dec 24;13:7933. doi: 10.1038/s41467-022-35504-x (PMC9789998; doi:10.1038/s41467-022-35504-x)
Supplement: Supplementary file 2 — Description of Supplementary Additional Files [file 41467_2022_35504_MOESM2_ESM.docx]

**Supplementary Video 1. The dynamic fusion of neighboring puncta.** The green spots represent the genome loci labeled and tracked by NFAT1-GFP whereas those red ones by nSIMBA.

**Supplementary Data 1. Calculated SNR values of SIMBA puncta.**

**Supplementary Data 2. nSIMBA regulates the NFAT1 related genes.**
