# Supplementary material for: Engineering inducible biomolecular assemblies for genome imaging and manipulation in living cells
Source: Nat Commun. 2022 Dec 24;13:7933. doi: 10.1038/s41467-022-35504-x (PMC9789998; doi:10.1038/s41467-022-35504-x)
Supplement: Supplementary file 6 — Reporting Summary [file 41467_2022_35504_MOESM6_ESM.pdf]

nature portfolio

Corresponding author(s): Yingxiao Wang
Last updated by author(s): Nov 26, 2022

Reporting Summary

Nature Portfolio wishes to improve the reproducibility of the work that we publish. This form provides structure for consistency and transparency in reporting. For further information on Nature Portfolio policies, see our Editorial Policies and the Editorial Policy Checklist.

Statistics

For all statistical analyses, confirm that the following items are present in the figure legend, table legend, main text, or Methods section.

n/a	Confirmed	
☐	☒	The exact sample size (*n*) for each experimental group/condition, given as a discrete number and unit of measurement
☐	☒	A statement on whether measurements were taken from distinct samples or whether the same sample was measured repeatedly
☐	☒	The statistical test(s) used AND whether they are one- or two-sided<br>*Only common tests should be described solely by name; describe more complex techniques in the Methods section.*
☒	☐	A description of all covariates tested
☐	☒	A description of any assumptions or corrections, such as tests of normality and adjustment for multiple comparisons
☐	☒	A full description of the statistical parameters including central tendency (e.g. means) or other basic estimates (e.g. regression coefficient) AND variation (e.g. standard deviation) or associated estimates of uncertainty (e.g. confidence intervals)
☐	☒	For null hypothesis testing, the test statistic (e.g. F, t, r) with confidence intervals, effect sizes, degrees of freedom and P value noted<br>*Give P values as exact values whenever suitable.*
☒	☐	For Bayesian analysis, information on the choice of priors and Markov chain Monte Carlo settings
☒	☐	For hierarchical and complex designs, identification of the appropriate level for tests and full reporting of outcomes
☒	☐	Estimates of effect sizes (e.g. Cohen's *d*, Pearson's *r*), indicating how they were calculated

Our web collection on statistics for biologists contains articles on many of the points above.

Software and code

Policy information about availability of computer code

Data collection	Time-lapse fluorescence images were acquired by MetaMorph 7.8.6.0 software (Molecular Devices, Sunnyvale, California). FRAP Data acquisition was done using NIS Elements AR 5.21.02 software.
Data analysis	All the fluorescence intensity measurements were quantified by Metafluor 7.10.4.450 software (Molecular Devices, Sunnyvale, CA) and ImageJ (2.3.0). All the plots were graphed in Graphpad Prism version 8. RNA-seq data were analyzed by softwares Ktrim v1.3.0, STAR v2.7.9a, featureCounts v2.0.3, DESeq2 v1.26.0, and databases JASPAR https://jaspar.genereg.net/, ENSEMBL gene annotation v101.

For manuscripts utilizing custom algorithms or software that are central to the research but not yet described in published literature, software must be made available to editors and reviewers. We strongly encourage code deposition in a community repository (e.g. GitHub). See the Nature Portfolio guidelines for submitting code & software for further information.

Data

Policy information about availability of data

All manuscripts must include a data availability statement. This statement should provide the following information, where applicable:

- Accession codes, unique identifiers, or web links for publicly available datasets
- A description of any restrictions on data availability
- For clinical datasets or third party data, please ensure that the statement adheres to our policy

Full data is available in the main text and supplementary materials. RNA-seq datasets are available at NCBI SRA with accession number PRJNA905219 (https://www.ncbi.nlm.nih.gov/sra/PRJNA905219). Source data are provided with this paper.

Human research participants

Policy information about studies involving human research participants and Sex and Gender in Research.

Reporting on sex and gender	No human research participants in this study.
Population characteristics	No human research participants in this study.
Recruitment	No human research participants in this study.
Ethics oversight	No human research participants in this study.

Note that full information on the approval of the study protocol must also be provided in the manuscript.

Field-specific reporting

Please select the one below that is the best fit for your research. If you are not sure, read the appropriate sections before making your selection.

[x] Life sciences [ ] Behavioural & social sciences [ ] Ecological, evolutionary & environmental sciences

For a reference copy of the document with all sections, see nature.com/documents/nr-reporting-summary-flat.pdf

Life sciences study design

All studies must disclose on these points even when the disclosure is negative.

Sample size	No sample-size calculation was performed. Sample sizes for experiments were determined based on similar published studies and to provide sufficient statistical power for data analysis (PMID: 30498221; PMID: 29566733; PMID: 35387989).
Data exclusions	No data were excluded from the analyses.
Replication	Attempts at replication were successful. The experiments were performed independently.
Randomization	Cells were randomized into different experimental groups (transfection, rapamycin stimulation, transduction).
Blinding	Investigators were not blinded to group allocation during data collection and analysis, which followed previous publications (PMID: 35387989).

Reporting for specific materials, systems and methods

We require information from authors about some types of materials, experimental systems and methods used in many studies. Here, indicate whether each material, system or method listed is relevant to your study. If you are not sure if a list item applies to your research, read the appropriate section before selecting a response.

Materials & experimental systems

n/a	Involved in the study
x	Antibodies
	x Eukaryotic cell lines
x	Palaeontology and archaeology
x	Animals and other organisms
x	Clinical data
x	Dual use research of concern

Methods

n/a	Involved in the study
x	ChIP-seq
	x Flow cytometry
x	MRI-based neuroimaging

Eukaryotic cell lines

Policy information about cell lines and Sex and Gender in Research

Cell line source(s)	HEK293T cell line was purchased from ATCC. U2OS cell line was a gift from Xavier Darzacq's lab (UC Berkeley)(it was generated by PMID: 15006351). 96-8 HCT116 cell line was a gift from Huimin Zhao's lab (UIUC) (PMID: 29912475).
Authentication	Cell lines were authenticated based on morphology, functionality and growth rates.
Mycoplasma contamination	All cell lines were tested negative for mycoplasma contamination.
Commonly misidentified lines (See ICLAC register)	No commonly misidentified lines used in this study.

Flow Cytometry

Plots

Confirm that:

- [x] The axis labels state the marker and fluorochrome used (e.g. CD4-FITC).
- [x] The axis scales are clearly visible. Include numbers along axes only for bottom left plot of group (a 'group' is an analysis of identical markers).
- [x] All plots are contour plots with outliers or pseudocolor plots.
- [x] A numerical value for number of cells or percentage (with statistics) is provided.

Methodology

Sample preparation	HEK 293T cells were transfected with the corresponding DNA constructs as described in manuscript. 48-72 hr post transfection, cells were trypsinized and resuspended in PBS at concentration of 1e6 - 3e6 per mL for FACS sorting.
Instrument	SONY SH800S cell sorter equipped with lasers of 488 nm, 561 nm, and 638 nm, using sorting chips of nozel size 100 µm (LE-C3210, SONY).
Software	Data collection: SH800 Cell Sorter Software version 2.1. Analysis: FlowJo 10.8.1.
Cell population abundance	The abundance of target cell population in the post-sort fraction was > 95%, as determined by the fluorescence of the transfected cells (e.g., mCherry).
Gating strategy	(1) Preliminary FSC-A/SSC-A gating for live cells: FSC-A (200K, 1.0M), SSC-A (100K, 1.5M). (2) FSC-W/SSC-A gating for single cells: FSC-A (200K, 1.0M), FSC-W (200,400). (3) fluorescence gating for target cells: fluorescence (1e4,1e6). See Supplementary Figure S13 for details.

- [x] Tick this box to confirm that a figure exemplifying the gating strategy is provided in the Supplementary Information.
